# Munc13-1 couples DAG and Ca^2+^ signaling to dynamic vesicle priming, synaptic short-term plasticity, and posttetanic potentiation

**DOI:** 10.1126/sciadv.aea0449

**Published:** 2026-02-13

**Authors:** Mrinalini Ranjan, Kun-Han Lin, Brian D. Mueller, Sonja M. Wojcik, Mareike Lohse, Thomas C. Südhof, Erik M. Jorgensen, Erwin Neher, Noa Lipstein, Nils Brose, Holger Taschenberger

**Affiliations:** ^1^ Department of Molecular Neurobiology, Max Planck Institute for Multidisciplinary Sciences, Göttingen, Germany.; ^2^ Göttingen Graduate School for Neurosciences, Biophysics, and Molecular Biosciences, Göttingen, Germany.; ^3^ Laboratory of Membrane Biophysics, Max Planck Institute for Multidisciplinary Sciences, Göttingen, Germany.; ^4^ Howard Hughes Medical Institute (HHMI), School of Biological Sciences, University of Utah, Salt Lake City, UT, USA.; ^5^ Leibniz-Forschungsinstitut für Molekulare Pharmakologie (FMP), Berlin, Germany.; ^6^ Department of Molecular and Cellular Physiology, Howard Hughes Medical Institute (HHMI), Stanford University School of Medicine, Stanford, CA, USA.; ^7^ Macau University of Science and Technology, Macau.; ^8^ Shenzhen Institute of Advanced Technology, Shenzhen, China.

## Abstract

Synaptic strength and plasticity are fine-tuned by neuromodulation and use-dependent second-messenger signaling. Presynaptic diacylglycerol (DAG), Ca^2+^, and Ca^2+^-calmodulin signaling converge on the essential synaptic vesicle (SV) priming protein Munc13-1 via its regulatory C_1_, C_2_B, and CaM-binding domains. Using brainstem-specific heterozygous mice expressing a DAG-binding-deficient Munc13-1 variant (Munc13-1^H567K^), we compared synaptic transmission in situ at glutamatergic calyx of Held synapses carrying either a single Munc13-1^H567K^ or a single Munc13-1^wt^ allele. Munc13-1^H567K/−^ synapses show enhanced initial strength but impaired steady-state release and slower recovery from depression. These deficits result from an increased initial abundance of fully primed SVs and a loss of activity-dependent acceleration of SV priming. Posttetanic potentiation (PTP) is strongly reduced in Munc13-1^H567K/−^ synapses and either increased or attenuated by C_2_B mutations that enhance or weaken Ca^2+^-phospholipid binding. Our data identify Munc13-1 as a target of presynaptic TrkB–phospholipase C–γ signaling and demonstrate that C_1_ and C_2_B domain-dependent regulation of Munc13-1 determines synaptic strength and shapes short-term plasticity and PTP.

## Teaser

Disabling Munc13-1 C_1_ domain signaling impairs synaptic strength, Ca^2+^-dependent vesicle priming, and posttetanic potentiation.

## INTRODUCTION

Action potential (AP)–induced transmitter release is temporally and spatially tightly controlled by local domains of elevated presynaptic Ca^2+^ concentration ([Ca^2+^]_i_) near open Ca^2+^ channels. Synaptic vesicles (SVs) undergo exocytosis via soluble NSF attachment protein receptor (SNARE)–mediated membrane fusion from a pool of SVs that are docked at presynaptic active zones (AZs) and primed for rapid membrane fusion upon Ca^2+^ influx (*1*, *2*). The formation of this pool of rapidly releasable SVs [readily releasable pool (RRP)] is governed by multiple soluble and AZ proteins that regulate reversible SV docking and priming (*3*, *4*), including proteins of the Munc13 (mammalian Unc13) family. At all conventional mammalian synapses tested thus far, Munc13s are strictly required for maintaining the RRP (*5*, *6*). Munc13s bridge SVs and plasma membranes (*7*, *8*) and regulate the conformation of the SNARE fusion protein syntaxin, thus promoting SNARE complex formation and close SV–plasma membrane contact (*9*–*11*). Super-resolution microscopy has shown that the number of Munc13-1 nanoclusters within AZs correlates with the number of functional SV release sites (*12*, *13*), supporting the idea that Munc13 assemblies define individual release sites.

Synapses must dynamically adjust the RRP replenishment rate to sustain high demands of SV consumption during periods of continuous AP firing and express diverse forms of activity-dependent short-term plasticity (STP), which determine neuronal circuit logic. Munc13s play a key role in the adjustment of synaptic efficacy and in shaping STP. Activity-dependent regulation of Munc13 priming activity is mediated by second messengers acting via two membrane-targeting modules, the DAG-binding C_1_ and the phospholipid-binding C_2_B domain, and via a Ca^2+^-CaM binding domain. The Munc13 C_1_ domain binds to the plasma membrane in a diacylglycerol (DAG)–dependent but Ca^2+^-independent manner, whereas the adjacent C_2_B domain binds phospholipids, predominantly PIP_2_, in a Ca^2+^-dependent but DAG-independent manner (*14*). In mammalian neurons, no presynaptic signaling pathway activating Munc13s via DAG has been identified, leaving the role of DAG-Munc13 signaling in intact circuits unclear.

DAG analogs, such as phorbol esters, potentiate transmitter release at many brain synapses (*15*–*17*). The loss of potentiation in cultured neurons expressing DAG/phorbol-ester-binding-deficient Munc13-1 identifies the Munc13 C_1_ domain as a principal DAG target in the presynapse (*18*, *19*), but the endogenous signaling pathways involved remain elusive and the molecular mechanisms of release potentiation are debated (*19*–*23*). Faster SV pool depletion during hyperosmotic stimulation of phorbol ester–treated synapses has been attributed to increased SV fusion probability (*21*, *24*). However, kinetic models based on a sequential two-step priming scheme (*25*) faithfully reproduce phorbol ester–induced release enhancement and STP changes by assuming little change in SV fusogenicity but strongly altered SV priming (*23*, *26*).

Tetanic high-frequency stimulation (THFS) of synapses can lead to a transient enhancement of transmitter release, which is similar in magnitude to DAG/phorbol-ester-induced potentiation and lasts up to several minutes (*27*–*31*). Like other forms of short-term synaptic enhancement (*32*), this posttetanic potentiation (PTP) is expected to modify information processing in neural circuits and is thought to sustain working memory (*33*, *34*). THFS increases global presynaptic [Ca^2+^]_i_ up to several micromolar (*27*, *29*) and may trigger DAG synthesis by activating Ca^2+^-dependent phospholipase Cs (PLCs) (*35*, *36*). Several Ca^2+^-dependent regulatory proteins were implicated in PTP induction, including Ca^2+^/CaM-dependent kinases (*37*–*39*) and protein kinase Cs (PKCs) (*28*, *40*, *41*). However, the precise role of kinase signaling remains unresolved as pharmacological and genetic perturbations targeting kinase signaling to interfere with PTP yielded contradictory results. PKC inhibitors have variable effects on the magnitude of PTP (*40*, *42*, *43*), and ablating PKC expression or disabling PKC phosphorylation by expression of mutant nonphosphorylatable target proteins attenuates PTP in some synapse types (*44*), whereas PTP persists in others (*45*, *46*).

To address these open questions, we used a combined genetic, electrophysiological, super-resolution imaging, and kinetic modeling approach and explored the role of the Munc13-1 C_1_ DAG-binding domain in regulating synaptic strength, STP, and PTP at calyx synapses in situ. We used a newly generated conditional Munc13-1 knockout mouse line (Munc13-1^fl/fl^) in combination with a mouse line carrying a mutation that disables C_1_ domain signaling (Munc13-1^H567K^; HK) (*18*). This allowed us to generate brainstem-specific heterozygous mice that carry either a single mutant allele (HK/−) or a single wild-type allele (wt/−) (*47*, *48*), circumventing perinatal lethality of homozygous Munc13-1^H567K/H567K^ mice (*18*).

We demonstrate that wt calyx AZs contain on average seven Munc13-1 nanoclusters and that both the amount and nanoscale organization of Munc13-1 are unaffected by the loss of one allele or by the H567K C_1_ domain mutation. TrkB receptor activation enhances glutamate release in wt/− but only very weakly in HK/− calyx synapses, identifying Munc13-1 as a downstream effector of presynaptic TrkB–PLC-γ signaling. HK/− calyx synapses exhibit increased synaptic strength despite unaltered presynaptic Ca^2+^ influx and Ca^2+^ influx–exocytosis coupling. The increased strength arises primarily from a greater abundance of fully primed SVs, whereas their fusogenicity remains nearly unchanged. In contrast, activity-dependent acceleration of SV pool replenishment is abolished by the H567K C_1_ domain mutation, resulting in reduced steady-state release during high-frequency stimulation. Numerical simulations based on a sequential two-step SV priming model (*25*) closely reproduce changes in synapse strength, STP, and recovery from depression in wt/− and HK/− calyx synapses. Gain-of-function and loss-of-function mutations in the C_1_ and C_2_B domains of Munc13-1 enhance and suppress PTP, respectively, demonstrating that Munc13-1–dependent SV priming is a key mechanism underlying PTP. Together, these findings highlight the crucial role of DAG-dependent and Ca^2+^-phospholipid–dependent regulation of Munc13-1 in controlling synaptic strength and fine-tuning activity-dependent modulation of STP.

## RESULTS

**Fig. 1.**
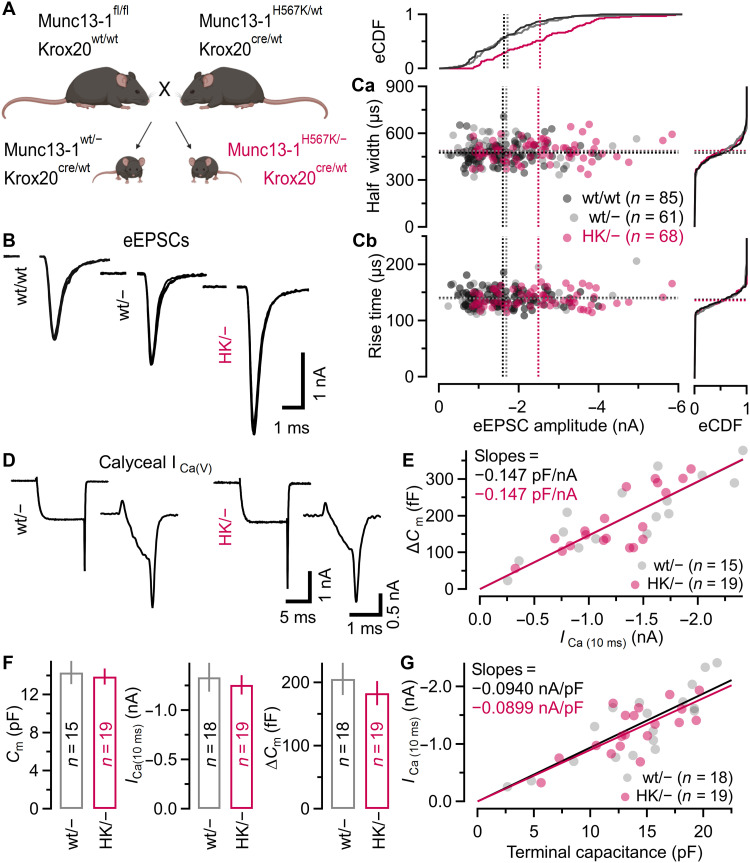
The Munc13-1 C_1_ domain H567K mutation increases eEPSC amplitudes but leaves eEPSC kinetics and presynaptic Ca^2+^ influx unaltered. (**A**) Mouse breeding scheme for generating brainstem-specific heterozygous conditional Munc13-1 knockout mice, which carry either a single Munc13-1^H567K^ mutant allele (HK/−) or a single Munc13-1^wt^ allele (wt/−) [created in BioRender, Ranjan, M. (2025) https://BioRender.com/vjevz5e]. (**B**) Unitary AP-evoked EPSCs recorded in a homozygous wt (wt/wt, left), a heterozygous wt (wt/−, middle), and a heterozygous mutant (HK/−, right) synapse in the presence of 1 mM kyn. Three consecutive eEPSCs are shown superimposed. (**C**) Scatterplots of eEPSC half-widths (Ca) and rise times (Cb) versus eEPSC amplitudes. eCDFs are shown on top and to the right. Mean values are indicated by the dotted horizontal and vertical lines. (**D**) Calyceal *I*_Ca(V)_ recorded in response to 10-ms step (top) or 1-ms step (bottom) depolarizations in a wt/− calyx (left) and an HK/− calyx (right). (**E**) Scatterplot of ∆*C*_m_ in response to 10-ms step depolarizations against the corresponding *I*_Ca(V)_ for wt/− and HK/− calyces. Solid lines represent linear regressions through the origin. Linear regression slopes for wt/− and HK/− calyces were identical. (**F**) Similar mean values of membrane capacitance (*C*_m_; left), *I*_Ca(V)_ (middle), and ∆*C*_m_ (right) in response to 10-ms step depolarizations in wt/− and HK/− terminals. Differences in mean values were not statistically significant (Welch’s *t* test). (**G**) Scatterplot of *I*_Ca(V)_ recorded in response to 10-ms step depolarizations against the terminal capacitance for wt/− and HK/− calyces. Linear regression slopes for wt/− and HK/− calyces were similar. Every circle in (C), (E), and (G) represents one individual synapse. The number of synapses tested is given in parentheses in (C), (E) to (G).

To assess the role of the Munc13-1 C_1_ domain in regulating SV priming, synaptic strength, STP, and PTP, we made use of a mouse line carrying a His^567^ to Lys (H567K) point mutation in the Cys_6_His_2_ motif, which disables the C_1_ domain and abolishes DAG binding (*49*). To circumvent perinatal lethality of homozygous Munc13-1^H567K/H567K^ mice (*18*), brainstem-specific heterozygous Munc13-1^H567K/−^ mice were generated by crossbreeding Munc13-1^H567K/wt^ mice with a newly generated Munc13-1^fl/fl^ mouse line (fig. S1A) and with Krox20^wt/Cre^ mice. Krox20^wt/Cre^ mice express Cre recombinase from embryonic day 17 (E17) in confined neuronal populations of the brainstem including the globular bushy cells of the anterior ventral cochlear nucleus (AVCN), which give rise to calyx of Held terminals contacting principal neurons (PNs) of the medial nucleus of the trapezoid body (MNTB). This genetic strategy enables the comparison of synaptic transmission in calyx synapses expressing either a single mutant (HK/−) or a single wild-type (wt/−) allele (Fig. 1A).

The loss of Munc13-1 expression after Cre-Lox recombination of the conditional Munc13-1 allele was validated by Western blot analysis of brains from E18 Munc13-1^−/−^ embryos, and there was no compensatory up-regulation of Munc13-2 (fig. S1B). Munc13-1 expression was reduced to 81.1 ± 3.3% in Munc13-1^wt/−^ brains (fig. S1C). Binding to the scaffold protein Rab3-interacting molecule (RIM) was not altered in Munc13-1^H567K^ (fig. S1D). Last, functional characterization of cultured hippocampal synapses prepared from Munc13-1^−/−^, Munc13-2^−/−^ (Munc13-1,2 DKO) E18 mice demonstrated the absence of an RRP and the lack of spontaneously occurring miniature excitatory postsynaptic currents (mEPSCs) (fig. S1, F and G).

### Increased glutamate release despite unaltered presynaptic Ca^2+^ influx in HK/− calyx synapses

We next compared synaptic properties of post–hearing onset (postnatal days 15 to 18) homozygous wild-type (wt/wt), heterozygous wild-type (wt/−), and heterozygous HK (HK/−) calyx of Held synapses by recording AP-evoked unitary EPSCs (eEPSCs) in voltage-clamped MNTB PNs. Unless noted, eEPSCs induced by afferent fiber stimulation were routinely recorded in the presence of 1 mM kynurenic acid (kyn) or 2 mM γ-D-Glutamylglycine (γ-DGG) to attenuate postsynaptic α-Amino-3-hydroxy-5-methyl-4-isoxazolepropionic acid receptor (AMPAR) desensitization and saturation. Mean eEPSC amplitudes were similar in wt/wt and wt/− calyx synapses but substantially larger in HK/− synapses (Fig. 1, B and C, and table S1), consistent with a right-shifted empirical cumulative distribution function (eCDF) of eEPSC amplitudes. Kinetic properties of eEPSCs were indistinguishable between all three genotypes (Fig. 1, B and C). Because mEPSC amplitudes (in the absence of kyn or γ-DGG) were similar in wt/wt, wt/−, and HK/− synapses (fig. S2, A and B, and table S1), the greater synaptic strength of HK/− synapses reflects increased glutamate release.

To test whether enhanced presynaptic Ca^2+^ influx accounts for the larger eEPSCs in HK/− synapses, we recorded pharmacologically isolated presynaptic voltage-gated Ca^2+^ currents [*I*_Ca(V)_] in voltage-clamped calyces. Membrane capacitance (*C*_m_), which is proportional to terminal size, as well as *I*_Ca(V)_ charges and amplitudes in response to 1- or 10-ms step depolarizations were similar in wt/− and HK/− calyces (Fig. 1, D to G, and table S1). Assaying glutamate release by measuring presynaptic membrane capacitance changes (∆*C*_m_) evoked by 10-ms step depolarizations revealed linear correlations between ∆*C*_m_ and *I*_Ca(V)_ (Fig. 1E) as well as between *I*_Ca(V)_ and *C*_m_ (Fig. 1G) among individual wt/− and HK/− terminals, reflecting the fact that larger calyces generate larger Ca^2+^ influx and larger exocytotic responses. The slopes of regression lines fitted to ∆*C*_m_ versus *I*_Ca(V)_ and *I*_Ca(V)_ versus *C*_m_ scatterplots were identical for both genotypes. Likewise, the dynamic modulation of *I*_Ca(V)_ amplitudes during short high-frequency trains was unaltered in HK/− calyces (fig. S2, C to E). Together, these data indicate that the HK C_1_ mutation does not affect presynaptic voltage-gated Ca^2+^ channel expression or function nor the Ca^2+^ influx–exocytosis coupling efficacy.

**Fig. 2.**
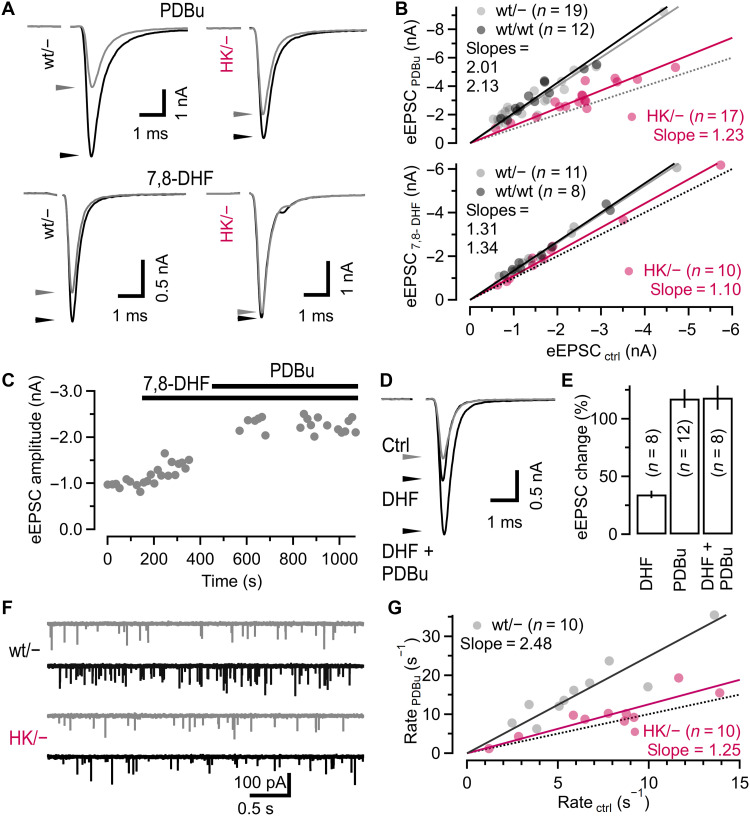
The H567K mutation of the Munc13-1 C_1_ domain impairs PDBu-induced and TrkB receptor agonist-induced potentiation of glutamate release. (**A**) eEPSCs recorded in the absence (gray) and presence (black) of 1 μM PDBu (top) or 20 μM 7,8-DHF (bottom) in wt/− (left) and HK/− (right) synapses. Waveforms represent averages of ≥3 consecutive eEPSCs. (**B**) Scattergraphs of eEPSC amplitudes measured in the presence and absence of PDBu (top) or 7,8-DHF (bottom) in the same wt/wt, wt/−, or HK/− synapses. Dotted and solid lines represent the identity lines and linear regressions through the origin, respectively. Regression slopes are a measure of eEPSC potentiation by PDBu or 7,8-DHF. (**C**) Unitary eEPSC amplitudes recorded under control conditions and in the presence of 7,8-DHF and PDBu plotted against time. Application intervals are indicated by the bars. Interval between consecutive eEPSCs ≥15 s. (**D**) Mean eEPSC waveforms under control conditions and during application of 7,8-DHF and 7,8-DHF + PDBu for the experiment shown in (C). (**E**) Mean changes in eEPSC amplitudes induced by application of 7,8-DHF (left), PDBu (middle), or 7,8-DHF + PDBu (right). Differences between PDBu and 7,8-DHF + PDBu were not statistically significant (Welch’s *t* test). (**F**) mEPSCs recorded in the absence of kyn before (gray) and after (black) 1 μM PDBu application in a wt/− (top) and an HK/− (bottom) synapse. (**G**) Scattergraphs of mean mEPSC rates in the presence and absence of PDBu in the same wt/− or HK/− synapses. Dotted and solid lines represent the identity line and linear regressions through the origin, respectively. Regression slopes are a measure for mEPSC rate potentiation by PDBu. Each circle in (B) and (G) represents an individual synapse. Numbers of synapses are given in parentheses in (B), (E), and (G).

DAG analogs such as phorbol 12,13-dibutyrate (PDBu) bind to the C_1_ domains of PKCs and Munc13s and enhance transmitter release (*15*, *50*, *51*). In cultured autaptic neurons homozygous for the DAG binding–deficient HK Munc13-1 variant, PDBu-induced eEPSC potentiation is strongly impaired (*18*). We therefore assessed PDBu-induced potentiation of eEPSCs amplitudes and mEPSC rates in wt/− and HK/− calyx synapses. Bath-applied PDBu increased eEPSCs in wt/wt and wt/− synapses on average about twofold but only ~1.23-fold in HK/− synapses (Fig. 2, A and B, top). The PDBu-induced potentiation of eEPSCs in wt/− synapses was mediated, in part, by an ~22% increased pool of fast-releasing SVs [*FRP* (*52*)] (fig. S2F) (*23*). In HK/− synapses, the *FRP* increased by only ~10% after PDBu application (fig. S2G). PDBu increased mEPSC frequencies more in wt/− (~2.48-fold) than in HK/− synapses (~1.25-fold; Fig. 2, F and G), similar to its effect on eEPSCs. To activate an endogenous intracellular signaling pathway that generates elevated presynaptic DAG levels (*53*), we bath applied the specific TrkB receptor agonist 7,8-dihydroxyflavone (7,8-DHF; 20 μM). 7,8-DHF increased eEPSC amplitudes in wt/wt and wt/− synapses by ~1.3-fold but only ~1.1-fold in HK/− synapses (Fig. 2, A and B, bottom). The residual DAG/PDBu-induced potentiation of eEPSC amplitudes and mEPSC rates in HK/− synapses likely reflects effects on targets other than Munc13-1, such as bMunc13-2, Munc13-3 (*54*), or PKCs (*19*, *28*, *55*). In wt/wt synapses, the potentiation of glutamate release by combined application of 7,8-DHF + PDBu was similar to the effect of PDBu alone, indicating a partial occlusion of PDBu-induced potentiation by 7,8-DHF (Fig. 2, C to E).

### Unperturbed subcellular Munc13-1 localization and copy numbers at AZs of HK/− calyx terminals

**Fig. 3.**
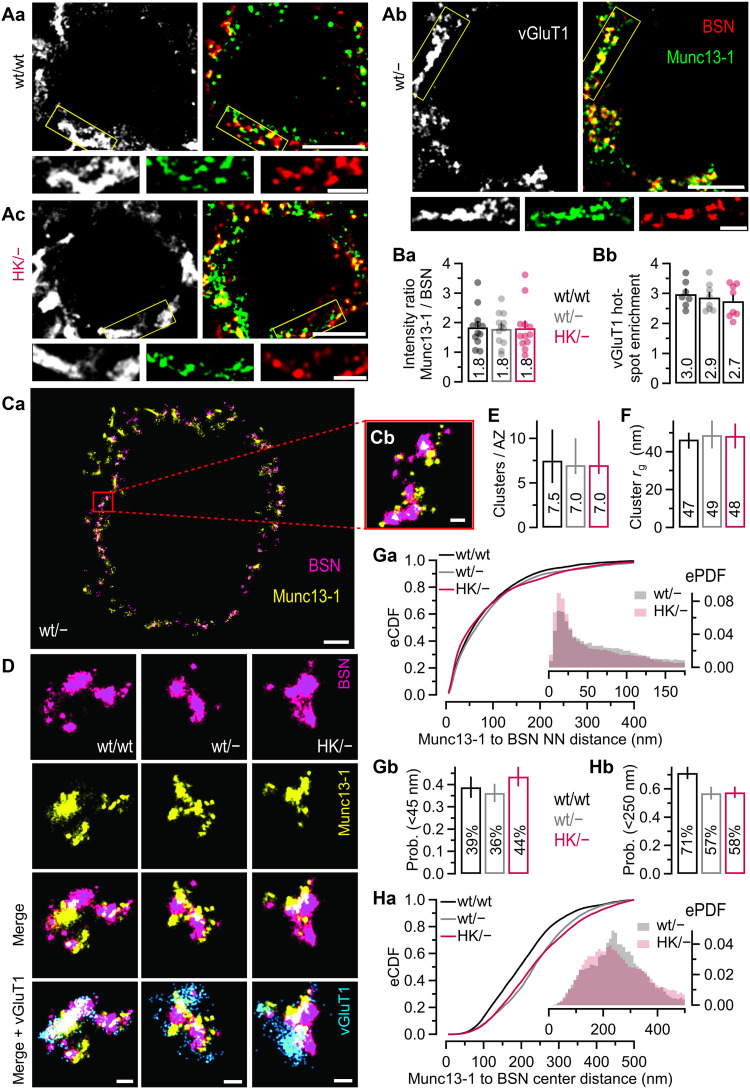
Munc13-1 expression at presynaptic AZs of calyx terminals is unchanged by gene dosage or the Munc13-1 HK C_1_ mutation. (**A**) Top: Confocal images showing labeling of vGluT1 (left) or Munc13-1 and BSN (right) for P18 wt/wt (Aa), wt/− (Ab), and HK/− (Ac) calyces. Bottom: Enlargements of yellow-framed areas. Scale bars, 5 μm (top) and 2 μm (bottom). (**B**) Munc13-1/BSN immunofluorescence intensity ratios in vGluT1-positive regions for wt/wt (12 ROIs; nine calyces), wt/− (12 ROIs; eight calyces), and HK/− (12 ROIs; nine calyces) (Ba) and hotspot enrichment of vGluT1 immunofluorescence intensity (90th percentile/median) for wt/wt (seven calyces), wt/− (eight calyces), and HK/− (eight calyces) (Bb). Bar graphs show means; scatter dot plots show individual ROIs (Ba) or calyces (Bb). (**C**) Single-molecule localization of BSN and Munc13-1 at a calyx identified by its ringlike morphology and by vGluT1 staining. (Ca) Single calyx terminal surrounding the postsynaptic cell body. Scale bar, 2 μm. Image was denoised and cropped to highlight putative AZs. (Cb) Expanded image of two putative AZs. Scale bar, 200 nm. (**D**) Comparison of putative AZs in wt/wt, wt/−, and HK/− calyces. Scale bars, 200 nm. (**E**) Median Munc13-1 cluster number per putative AZ. (**F**) Median radius of gyration (*r*_g_) of Munc13-1 clusters. (**G**) NNDs between Munc13-1 and BSN particles for wt/wt, wt/−, and HK/− calyces. (Ga) eCDFs and corresponding ePDFs (inset). (Gb) Cumulative probability of NNDs < 45 nm. (**H**) Distances of Munc13-1 particles to nearest BSN cluster centers for wt/wt, wt/−, and HK/− calyces. (Ha) eCDFs and corresponding ePDFs (inset). (Hb) Cumulative probability of distances < 250 nm. Numbers indicate means [(B), (Gb), and (Hb)] or medians [(E) and (F)]. Error bars indicate bootstrapped 95% confidence intervals [(E) and (F)] or SEM [(Gb) and (Hb)]. Sample sizes (calyces/AZs) for (E) to (H): wt/wt, 5/24; wt/−, 6/21; HK/−, 4/27.

To explore whether changes in Munc13-1 abundance at AZs can account for increased synaptic strength in HK/− calyx synapses, brain cryosections containing the MNTB region were obtained from P18 mice and triple immunostained for Munc13-1, the AZ marker protein bassoon (BSN), and the vesicular glutamate transporter vGluT1 (Fig. 3, A and B). vGluT1 immunofluorescence served as a marker to discriminate glutamatergic calyces from small inhibitory glycinergic boutons contacting MNTB PNs. Colabeling of BSN and vGluT1, therefore, allowed the identification of Munc13-1–positive AZs in calyces (Fig. 3A). Quantification of Munc13-1 immunofluorescence intensity relative to that of BSN in vGluT1-positive regions of interest (ROIs) revealed no difference between wt/wt, wt/−, and HK/− calyces (Fig. 3Ba), indicating that neither gene dosage nor the HK C_1_ mutation affect the amount of Munc13-1 at AZs. Hotspot enrichment of vGluT1 immunofluorescence intensity (90th percentile/median) was ~3-fold for all genotypes (Fig. 3Bb). Because this parameter quantifies accumulation of vGluT1, we conclude that the spatial clustering of glutamatergic SVs within calyces is unaltered.

Next, we examined whether AZ organization was perturbed in HK/− calyces. The spatial relationships and nanometer-scale organization of Munc13-1 in calyces of brainstem cryosections triple-immunolabeled for BSN, Munc13-1, and vGluT1 were examined by stochastic optical reconstruction microscopy (STORM) followed by DBScan cluster analysis (*56*) (Fig. 3, C and D). To assess whether AZ topology differs between genotypes, the number and size of BSN and Munc13-1 nanoclusters were determined at vGluT1-positive AZs. The median number of BSN (~3) and Munc13-1 (~7.2; Fig. 3E) nanoclusters per AZ was similar in all genotypes. The size of BSN and Munc13-1 clusters was quantified by measuring the radius of gyration (*r_g_*). The median *r_g_* was larger for BSN (~118 nm) than for Munc13-1 (~48 nm; Fig. 3F) nanoclusters, with no differences between genotypes.

Nearest-neighbor analysis was used to assess the spatial distribution of Munc13-1 molecules relative to the nearest BSN molecule. Empirical probability density functions (ePDFs; Fig. 3Ga, inset) of nearest-neighbor distances (NNDs) revealed a narrow (~20 nm wide) peak at ~25-nm NND for wt/− and HK/− terminals, indicating unperturbed Munc13-1 interactions with AZ components (*3*). Cumulative probability analysis showed similar proportions of short-range (<45 nm) and intermediate-range (45 to 500 nm) NNDs across all genotypes. Short-range NNDs accounted for about 35 to 45% of the NNDs (Fig. 3Gb). ePDFs of Munc13-1 distances to the center of the nearest BSN nanocluster exhibited a broad peak between ~100 and 300 nm (Fig. 3Ha, inset), indicating that Munc13-1 molecules tend to be located laterally to BSN in all genotypes (Fig. 3H). The small shifts between Munc13-1–to–BSN-center distributions are within the uncertainty of the measurement due to the linkage errors associated with indirect immunolabeling (*57*, *58*).

### A higher abundance of fully primed SVs accounts for increased glutamate release in HK/− calyx synapses

**Fig. 4.**
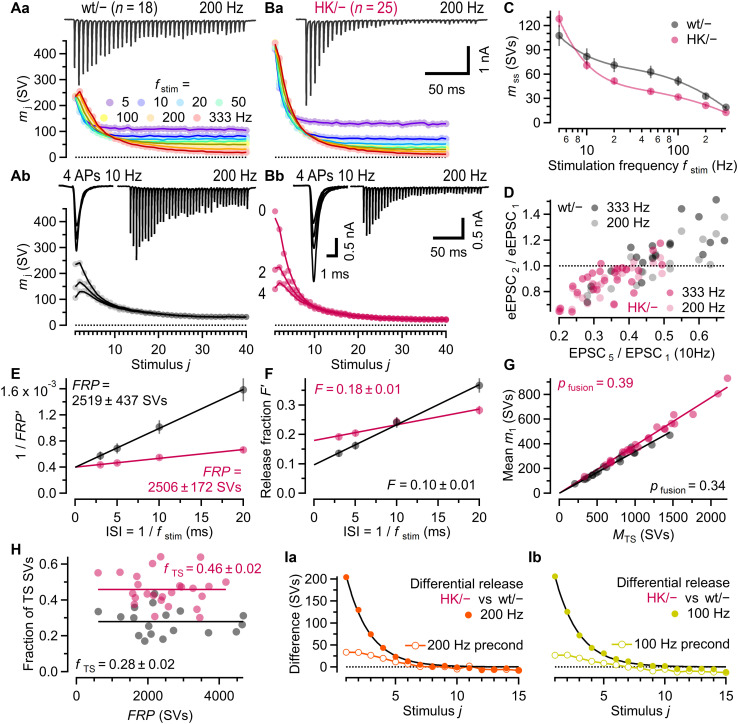
The HK C_1_ mutation alters STP during stimulus trains. (**A**) Mean quantal content (*m*_j_) plotted against stimulus number *j* (bottom) for regular eEPSC trains (*f*_stim_ = 5 to 333 Hz; Aa) or preconditioned (two or four stimuli, 10 Hz) 100- and 200-Hz eEPSC trains (Ab) in wt/− synapses. Top: Sample 200-Hz eEPSC train [(Aa) and (Ab), right]. Sample eEPSCs during 10-Hz preconditioning are shown superimposed [(Ab), left]. Data for 200-Hz trains without preconditioning are replotted in (Ab). (**B**) Similar experiments as in (A), but in HK/− synapses. The number of synapses is given in parentheses in (A) and (B). (**C**) Mean steady-state quantal content (*m*_ss_; mean eEPSCs_35_ to eEPSC_40_) plotted against *f*_stim_. Sample sizes as in (Aa) and (Ba). Lines represent smoothing spline fits. (**D**) 333- and 200-Hz *PPR*s plotted against 10-Hz depression ratios (eEPSC_5_/eEPSC_1_) in the same synapses. (**E**) Mean 1/*FRP*′ plotted versus the reciprocal of *f*_stim_ at which *FRP*′ was measured. Intersections of regression lines estimate 1/*FRP* corrected for incomplete pool depletion. (**F**) Mean release fraction *F*′ (eEPSC_1_/FRP′) plotted versus the reciprocal of *f*_stim_ at which *F*′ was measured. Intersections of regression lines estimate *F* corrected for incomplete pool depletion. (**G**) Scatterplot of the initial quantal content (*m*_1_) against preexisting TS pool size (*M*_TS_) from NTF decomposition for individual wt/− and HK/− synapses. Slopes of regression lines through the origin estimate *p*_fusion_. (**H**) Scatterplot of *f*_TS_ versus *FRP* size. Solid lines represent mean *f*_TS_ values. (**I**) Mean differences in *m*_j_ between HK/− and wt/− synapses plotted against *j* for 200-Hz (Ia) and 100-Hz (Ib) trains, without (filled circles) and with (empty circles) preconditioning (four stimuli, 10 Hz). Black lines fit difference traces without preconditioning using a geometric series. Error bars indicate SEM. Error estimates in (E) and (F) were obtained using a balanced bootstrap.

To assess HK C_1_ mutation–induced changes in STP, eEPSC trains were recorded in response to a wide range of stimulation frequencies (*f*_stim_) [1 to 2 Hz (15 stimuli) and 5 to 333 Hz (40 stimuli)] in wt/− and HK/− calyx synapses (Fig. 4, A and B). eEPSC amplitudes were converted to quantal content (*m*) using an effective quantal size (q^*^) of −6.6 pA (*23*, *25*). Mean release time courses of wt/− synapses exhibited frequency-dependent steady-state depression and transient eEPSC net facilitation for *f*_stim_ ≥ 200 Hz (Fig. 4Aa). HK/− synapses lacked net facilitation at all *f*_stim_ (Fig. 4Ba). Brief 10-Hz preconditioning (two or four APs) just before high-frequency stimulation largely eliminated the differences in high-frequency eEPSC trains between wt/− and HK/− synapses (Fig. 4, Ab and Bb). Steady-state quantal content (*m*_ss_) was increased for *f*_stim_ = 5 Hz but reduced for all *f*_stim_ ≥ 10 Hz in HK/− synapses (Fig. 4C). Differences in STP patterns between HK/− and wt/− synapses were evident not only in averaged data but also when plotting individual 200- and 333-Hz *PPR*s (eEPSC_2_/eEPSC_1_) against 10-Hz steady-state depression ratios (EPSC_5_/EPSC_1_; Fig. 4D) from the same synapses. A majority of wt/− synapses exhibited *PPR*s > 1 and only moderate depression upon 10-Hz stimulation, whereas nearly all HK/− synapses had *PPR*s < 1 and stronger depression upon 10-Hz stimulation.

We next derived *FRP* estimates for wt/− and HK/− synapses based on the cumulative quantal release during 50-, 100-, 200-, and 333-Hz eEPSC trains. Mean *FRP* estimates obtained at any single *f*_stim_ (*FRP*′), which are compromised by incomplete SV pool depletion, were consistently larger in HK/− as compared to wt/− synapses. Differences in *FRP*′ estimates between wt/− and HK/− synapses diminished with increasing *f*_stim_ (Fig. 4E). After correction for incomplete SV pool depletion as described previously (*23*, *25*, *59*), mean *FRP* estimates were similar in wt/− and HK/− synapses (table S1), which was corroborated by presynaptic ∆*C*_m_ recordings (fig. S3). Among individual wt/− and HK/− synapses, *FRP* estimates varied ~7.6-fold (604 to 4609 SVs) and ~6.9-fold (603 to 4184 SVs), respectively (Fig. 4H).

The release fraction *F* = eEPSC_1_/*FRP*, often designated as vesicular release probability (*p*_vr_), represents the fraction of *FRP* SVs released by a single presynaptic AP. The mean *F* estimate was ~1.86-fold higher in HK/− synapses (Fig. 4F). If the *FRP* consisted of a single pool of functionally homogeneous fusion-competent SVs, then *F* would correspond to the fusion probability (*p*_fusion_) of *FRP* SVs. However, the intricate details of STP patterns at brain synapses are difficult to reproduce by simple single-pool models. Accordingly, multiple functionally distinct SV subpools are often postulated (*25*, *26*, *31*, *60*–*63*), each of which may be largely depleted by strong stimulation, such that standard protocols for assaying pool size measure the sum of all subpools. A two-step kinetic priming scheme was recently proposed (*25*, *64*), which recapitulates the sequential release machinery assembly. This model defines the *FRP* as a dynamic equilibrium of SVs in two priming states, tightly (TS) or loosely (LS) docked to the AZ membrane (*FRP* = LS + TS), with only TS SVs being fully primed and fusion competent. Accordingly, the release probability for *FRP* SVs is determined by the compound probability of two independent events occurring together: (i) an *FRP* SV being in the TS state at AP arrival [*f*_TS_ = TS/(LS + TS) = TS/*FRP*] and (ii) a TS SV fusing in response to the AP. Hence, the release fraction *F* reflects the product *f*_TS_ · *p*_fusion_.

Estimates for *p*_fusion_, LS, and TS SV subpool sizes and *f*_TS_ were obtained by subjecting all 5- to 333-Hz eEPSC trains to nonnegative tensor factorization (NTF). As adapted to the analysis of trains of synaptic responses (*65*), NTF decomposes such trains into kinetic components contributed by SVs residing in distinct priming states before stimulation (*23*, *25*). NTF-decomposition fits described wt/− and HK/− eEPSC trains well, with little deviation from experimental data (Fig. 4, A and B). NTF-derived estimates for the number of preexisting TS SVs (*M*_TS_) varied ~7.1-fold among wt/− synapses (205 to 1457 SVs) and ~6.7-fold among HK/− synapses (332 to 2217 SVs). Scatterplots of initial quantal content (*m*_1_) against *M*_TS_ (Fig. 4G) showed a strong linear correlation, reflecting the NTF constraint of similar initial *p*_fusion_ among synapses of a given genotype and experimental condition. The slopes of linear regressions through the origin represent the initial *p*_fusion_ (wt/−: ~0.34, HK/−: ~0.39). Similar *p*_fusion_ estimates were obtained from *PPR*s and steady-state depression during 10-Hz eEPSC trains [eqn. 31 in Lin, Taschenberger, and Neher (*25*)], whether based on averaged data (wt/−: ~0.34; HK/−: ~0.39) or individual synapses (wt/−: 0.35 ± 0.02; HK/−: 0.41 ± 0.02). Whereas *p*_fusion_ increased by only ~15% in HK/− synapses, the fraction of TS SVs (*f*_TS_) was on average ~1.64-fold higher (Fig. 4H). Thus, the higher *F* estimate for HK/− synapses is predominantly attributable to changes in the LS-TS priming equilibrium (wt/−: *F* = 0.10 ≈ 0.28 · 0.34; HK/−: *F* = 0.18 ≈ 0.46 · 0.39).

If enhanced release during high-frequency trains in HK/− synapses is primarily caused by a higher contribution of rapidly consumed TS SVs, we can isolate that additional TS release component by subtracting nonconditioned high-frequency eEPSC trains recorded in wt/− from those recorded in HK/− synapses. The differential release quickly decays to close to zero within 5 to 10 stimuli and can be fitted by a geometric series 𝑚_𝑗_ = 𝑚_1_ · (1 − 𝑝_*fusion*_)^𝑗−1^, where *j* denotes stimulus 1 to 15, and *m*_j_ denotes the differences in quantal content between HK/− and wt/− synapses at stimulus *j* (200 Hz, Fig. 4Ia; 100 Hz, Fig. 4Ib). This provides an independent estimate for *p*_fusion_ of ~0.40, which is close to the NTF-derived *p*_fusion_ for HK/− synapses.

### The accelerated eEPSC recovery following high-frequency stimulation is abolished in HK/− synapses

**Fig. 5.**
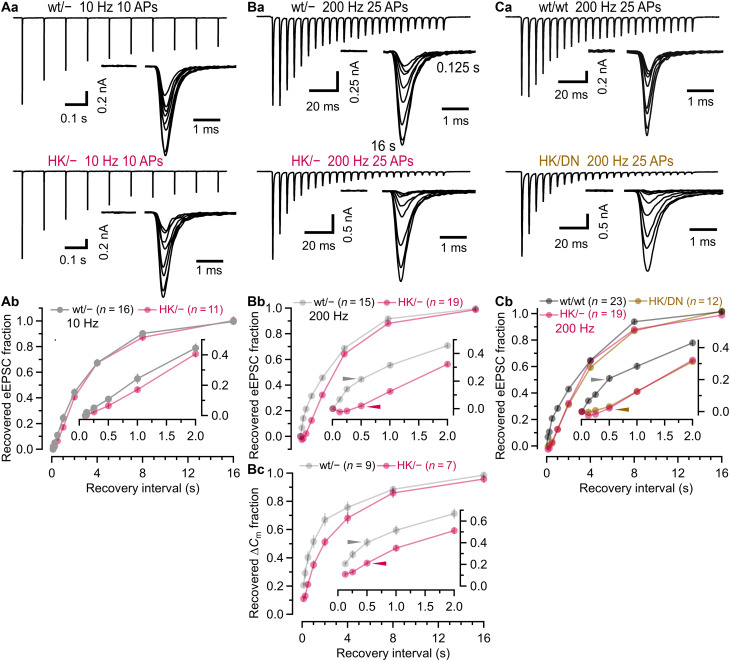
HK/− calyx synapses lack the fast component of eEPSC recovery after high-frequency stimulation, whereas recovery following low-frequency stimulation is unperturbed. (**A**) SV pool depletion was induced by 10-Hz stimulation (10 APs), and eEPSC recovery (eEPSC_test_) was measured at intervals of 0.125, 0.25, 0.5, 1, 2, 4, 8, and 16 s. Sample 10-Hz eEPSC trains (Aa, left) and eEPSC_test_ (Aa, right) at varying recovery intervals are shown for a wt/− (Aa, top) and an HK/− (Aa, bottom) synapse. (Ab) Mean recovery time courses after 10-Hz stimulation. Inset: First 2 s of recovery at an expanded timescale. (**B**) Similar experiment as in (A) using 200-Hz trains (25 APs) (Ba). (Bb) Mean recovery time courses after 200-Hz stimulation. Inset: Initial 2 s of recovery at an expanded timescale. (Bc) Mean SV pool recovery time course measured by presynaptic ∆*C*_m_ in wt/− and an HK/− calyces. Step depolarizations (3 ms to 0 mV) were applied to largely deplete the fast-releasing SV pool (*FRP*). Recovery was assessed by ∆*C*_m_ responses to a second 3-ms step at varying intervals. (**C**) Similar experiment as in (A) but using wt/wt and trans-heterozygous HK/DN synapses (Ca). (Cb) Mean recovery time courses after 200-Hz stimulation. Inset: Initial 2 s of recovery at an expanded timescale. eEPSC recovery in HK/− is shown superimposed. Fractional recovery did not differ between HK/DN and HK/− synapses (*P* > 0.05; Tukey’s multiple comparisons of means) but was significantly faster in wt/− and wt/wt synapses for all intervals ≤2 s (*P* < 0.0001; Tukey’s multiple comparisons of means). Numbers of synapses or terminals are indicated in (Ab), (Bb), (Bc), and (Cb). Arrowheads in (Bb), (Bc), and (Cb) mark differences at the 0.5-s recovery interval. Error bars indicate SEM.

Lower *m*_ss_ during high-frequency stimulation of HK/− synapses is indicative of impaired SV pool replenishment. We therefore compared eEPSC recovery time courses after low-frequency (10 Hz, Fig. 5A; 20 Hz, fig. S4) and high-frequency (100 Hz, fig. S5Cb; 200 Hz, Fig. 5B) stimulation by plotting the recovered eEPSC fractions defined as (eEPSC_test_ − eEPSC_ss_)/(eEPSC_1_  − eEPSC_ss_) against recovery intervals. eEPSC recovery after low-frequency stimulation followed a similar, single-exponential time course in HK/− and wt/− synapses (Fig. 5A and fig. S4). In contrast, high-frequency stimulation elevates presynaptic global [Ca^2+^]_i_ by summating individual AP-evoked Ca^2+^ transients and leads to biphasic recovery from depression at calyx and other brain synapses (*66*–*69*). Intriguingly, the rapid component of eEPSC recovery observed during the initial 500 ms after high-frequency stimulation in wt/− synapses was completely absent in HK/− synapses (Fig. 5B and fig. S5Cb). After long recovery intervals of ≥16 s, eEPSCs had fully recovered in both wt/− and HK/− synapses. To more directly assess the SV pool replenishment kinetics after strong presynaptic stimulation, we compared the presynaptic ∆*C*_m_ measured in response to a 3-ms step depolarization, which is thought to primarily deplete the *FRP* (*70*), with a second ∆*C*_m_ response measured at variable recovery intervals. The resulting time courses of the ratios of the second over the first ∆*C*_m_ response confirm slower SV pool refilling kinetics in HK/− calyces (Fig. 5Bc).

The reduced *m*_ss_ and slower recovery from SV pool depletion are reminiscent of functional deficits in calyx synapses expressing a Munc13-1 variant carrying two point mutations that impair Ca^2+^-dependent phospholipid binding to the C_2_B domain (Munc13-1^DN/DN^; DN/DN) (*59*). Considering that (i) steady-state release during and eEPSC recovery after high-frequency stimulation, which are both regulated by presynaptic [Ca^2+^]_i_ (*59*, *66*, *71*), are similarly reduced in DN/DN and HK/− synapses, that (ii) PDBu-induced eEPSC potentiation is unaltered in DN/DN calyx synapses (fig. S6), and that (iii) DAG/PDBu binding to the Munc13-1 C_1_ occurs independently of [Ca^2+^] (*14*), the comparable effects of HK C_1_ and DN C_2_B mutations suggest that (i) activity-dependent enhancement of SV priming activity is primarily mediated by the Munc13-1 C_2_B sensing elevated [Ca^2+^]_i_ and (ii) the HK Munc13-1 C_1_ mutation perturbs the function of the adjacent C_2_B (*72*–*74*). To corroborate this notion, we generated trans-heterozygous Munc13-1^HK/DN^ (HK/DN) mice by crossbreeding heterozygous HK/wt and DN/wt parents. Heterozygous Munc13-1 wt/− calyx synapses show normal eEPSC recovery from depression (compare Fig. 5B and Fig. 5C), and heterozygous Munc13-1 synapses (DN/wt or HK/wt) generally show normal transmission (*5*, *18*). If Munc13-1 C_1_ and C_2_B domains independently supported activity-dependent acceleration of SV priming, trans-heterozygous Munc13-1^HK/DN^ (HK/DN) synapses would be expected to show little functional deficits. However, the steady-state release during 200-Hz trains and the fast component of eEPSC recovery from depression were similarly reduced in HK/DN (Fig. 5C) and DN/DN (*59*) synapses, which is consistent with the notion of an intramolecular interdependence between the C_1_ and C_2_B domains. This result suggests that the HK C_1_ mutation slows eEPSC recovery by perturbing the function of the adjacent C_2_B domain.

### A kinetic two-step priming model reproduces STP and eEPSC recovery from depression in wt/− and HK/− calyx synapses

**Fig. 6.**
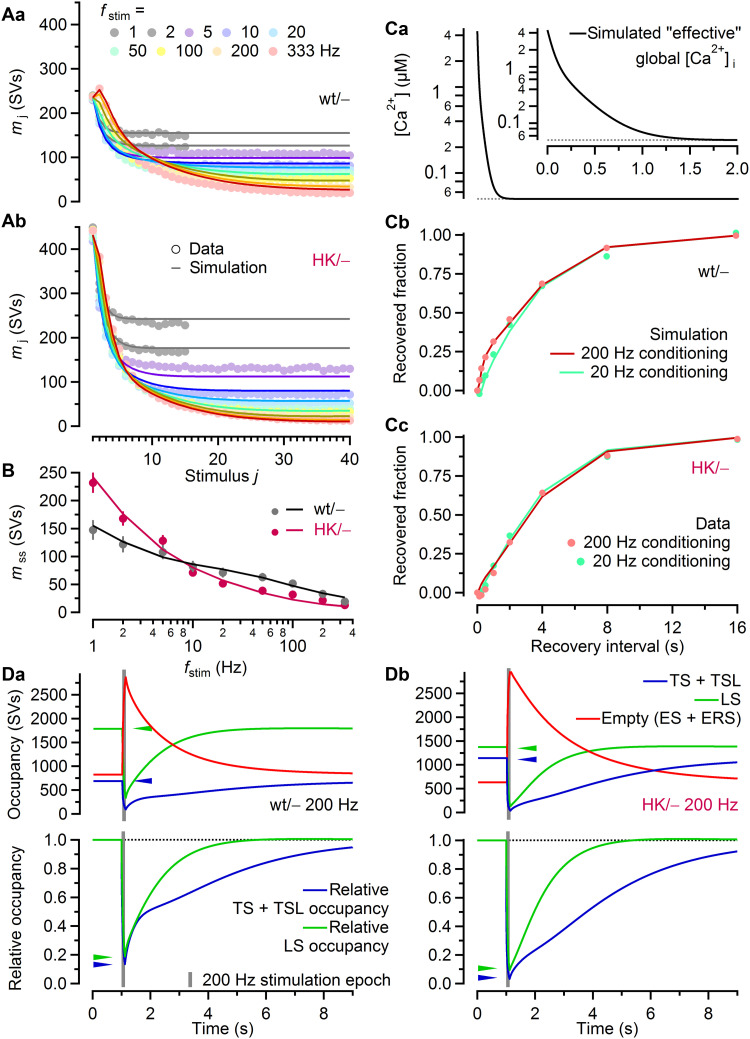
A two-step SV priming and fusion scheme reproduces experimentally observed STP and eEPSC recovery time courses in response to train stimulation in wt/− and HK/− calyx synapses. (**A**) Model predictions for the mean quantal content during stimulus trains (*m*_j_; 40 APs; lines) superimposed on experimental data (circles; replotted from Fig. 4, Aa and Ba) for wt/− (Aa) and HK/− (Ab) synapses. Gray circles and lines represent 1- and 2-Hz data and simulations. The remaining frequencies are color coded as in Fig. 4 (Aa and Ba). (**B**) Model predictions for mean steady-state release (*m*_ss_; 40 APs; lines) superimposed onto experimental data (circles; replotted from Fig. 4C) for wt/− and HK/− synapses. (**C**) Predicted eEPSC recovery time courses after 200-Hz (dark red lines, 25 APs) or 20-Hz (bright teal lines, 10 APs) stimulation in wt/− (Cb) and HK/− (Cc) synapses. Experimental data are superimposed (circles). The same simulated global Ca^2+^ transient (Ca) was used for both genotypes (0 s: onset of recovery). Inset: First 2 s of the global Ca^2+^ transient during eEPSC recovery at an expanded timescale. (**D**) Top: Simulated time courses of occupancy of LS (not fusion-competent) and TS + TSL (fusion-competent) SV subpools during and after 200-Hz stimulation (25 APs) in wt/− (Da) and HK/− (Db) synapses. The simulated time courses of the number of empty sites (ES + ERS) are shown for comparison. Bottom: Same simulation as above, but LS and TS + TSL occupancies are normalized to their respective initial resting values. The recovery time course of the LS subpool is largely preserved in HK/− synapses, whereas the recovery of fusion-competent SVs is delayed. Arrowheads in (D) mark the initial (top) and minimum (bottom) LS and TS + TSL occupancies.

We next explored whether increased synaptic strength and aberrant STP during stimulus trains in HK/− synapses can be reproduced by numerical simulations based on the recently proposed two-step LS-TS priming model (*23*, *25*, *26*, *60*, *64*). To model the AP-induced “effective” intracellular Ca^2+^ transient, we used a combination of a local and a global component as previously described (*23*). The number of LS and TS SVs and the value for *p*_fusion_ were constrained to NTF-derived estimates, and parameters determining the dynamics of *p*_fusion_ during stimulus trains were similar to previous studies (*23*, *25*). The remaining model parameters were optimized by trial and error to closely reproduce experimentally observed release time courses (Fig. 6, A and C) and *m*_ss_ (Fig. 6B) for *f*_stim_ ranging from 1 to 333 Hz.

Synaptic strength and STP in HK/− synapses were faithfully reproduced after setting initial *p*_fusion_ values to NTF-derived estimates and choosing values for the rate constant of the forward (*k*_1,rest_ and *k*_2,rest_) and backward (*b*_1_ and *b*_2_) state transitions at resting [Ca^2+^]_i_ in such a way that the NTF-derived estimates for the number of LS and TS SVs in resting synapses were matched. To achieve a good fit, the model parameters *k*_1,rest_, *b*_1_, and *b*_2_ and the total number of release sites (*N*_total_) required only marginal or no adjustments. However, the LS→TS transition rate constant at resting [Ca^2+^]_i_ (*k*_2,rest_) had to be increased substantially (~2.15-fold) for simulated HK/− synapses to account for their shifted LS-TS balance at rest [*f*_TS_ = *k*_2,rest_/(*k*_2,rest_  + *b*_2_); table S2]. Furthermore, the model parameters σ_1_ and σ_2_, which define the Ca^2+^ sensitivity of the ES→LS and LS→TS transitions, respectively, were adjusted to account for the changes in the relationship between *m*_ss_ and *f*_stim_ (table S2).

eEPSC recovery after 20-Hz stimulation was well reproduced by the model for both genotypes. Moreover, the model predicted a reduced Ca^2+^-dependent acceleration of eEPSC recovery following 200-Hz stimulation for HK/− synapses (Fig. 6C). The reduced sensitivity of the ES→LS transition to elevated [Ca^2+^]_i_ levels (σ_1_) leads to stronger depletion of LS and TS subpools in simulated HK/− synapses at high stimulation frequencies. Consequently, steady-state release is lower despite slightly higher *p*_fusion_, and eEPSC recovery is slower. Figure 6D illustrates, for both genotypes, the predicted occupancy of LS and TS + TSL subpools in comparison to the number of empty release sites (ES + ERS) at rest, during 200-Hz stimulation, and during recovery from pool depletion. The number of fusion-competent SVs (TS + TSL) decreases more strongly in simulated HK/− synapses during 200-Hz stimulation (Fig. 6D). Whereas the LS subpool recovers with similar kinetics in simulated wt/− and HK/− synapses, the TS subpool recovers more slowly in simulated HK/− synapses, consistent with experimental observations (Figs. 5Bb and 6C).

Both phorbol ester application and the Munc13-1 H567K mutation enhance the release fraction *F*, which originally led to the conclusion that this mutation mimics the C_1_ domain-activated state (*21*). Consistent with this view, elevated values of the model parameters governing the LS→TS transition at rest and during activity (*k*_2,rest_ and σ_2_) are required to reproduce PDBu-induced STP changes at calyx synapses (*23*). However, in contrast to modeling PDBu-induced enhancement, which does not require a change in σ_1_, reproducing STP in HK/− synapses necessitates a strong reduction in σ_1_. This distinction is consistent with the different effects of PDBu application and the Munc13-1 H567K mutation on steady-state transmission and eEPSC recovery kinetics (fig. S5, A to D).

### Severely reduced PTP in HK/− calyx synapses

**Fig. 7.**
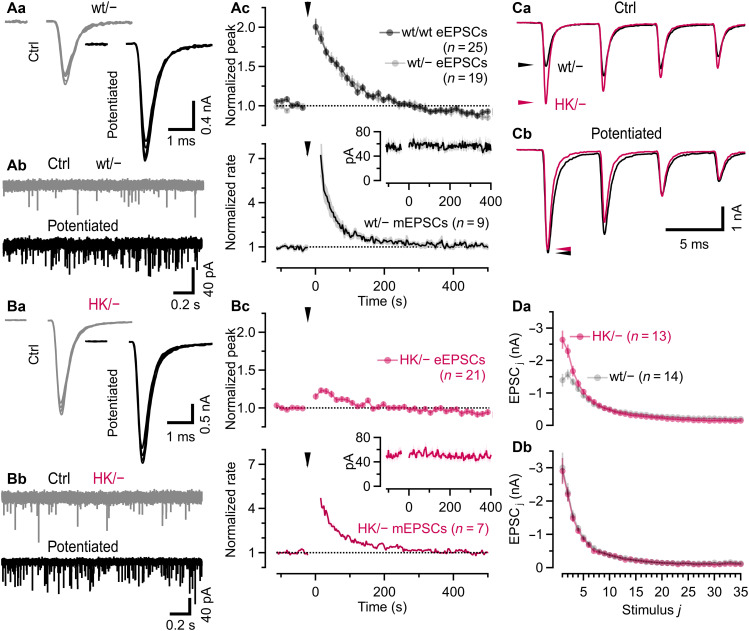
HK/− calyx synapses exhibit strongly impaired PTP of eEPSC amplitudes. (**A**) eEPSCs (Aa; five consecutive eEPSCs; 1 mM kyn) and mEPSC (Ab; no kyn) recorded before (gray) and after (black) PTP induction by THFS (8 s, 200 Hz) in wt/− synapses. eEPSC amplitudes were measured every 15 s before and after THFS, normalized to the mean value before THFS, and plotted over time (Ac, top) for wt/wt and wt/− synapses. mEPSC rate potentiation was quantified from continuous recordings by calculating mean event rates over nonoverlapping 2-s bins (Ac, bottom). The arrowheads indicate the THFS onset, and *t* = 0 s marks the end of the tetanus. Inset: Time course of mean mEPSC amplitudes. (**B**) Similar experiments as in (A) but for HK/− synapses. The PTP of eEPSC amplitudes is strongly reduced [(Bc), top], whereas mEPSC rate potentiation is less affected [(Bc), bottom]. Inset: Time course of mean mEPSC amplitudes. (**C**) eEPSC trains evoked by 200-Hz stimulation (35 APs) before [(Ca), ctrl] and 15 s after THFS (Cb, potentiated) in a wt/− and an HK/− synapse. Only the first four eEPSCs are shown. Arrowheads mark eEPSC_1_ amplitudes. (**D**) Mean eEPSC train amplitudes before (Da) and after (Db) PTP induction for wt/− and HK/− synapses. Error bars [(Ac), top; (Bc), top; and (D)] and shaded regions [(Ac), bottom and (Bc), bottom] represent SEM. Numbers of synapses are given in parentheses in (Ac), (Bc), and (D).

An increased abundance of so-called “superprimed” SVs has been proposed as a common mechanism underlying both PTP and PDBu-induced eEPSC potentiation (*31*). Given the reduced sensitivity of HK/− calyx synapses to PDBu, we next tested whether they also exhibit diminished PTP. Subjecting wt/wt and wt/− synapses to THFS (200 Hz, 8 s, 1600 APs) resulted in an ~2.01 ± 0.08-fold (*n* = 41) and ~2.08 ± 0.08-fold (*n* = 26) eEPSC potentiation, which decayed to half-maximum potentiation (*t*_0.5_) within ~66 and ~64 s (Fig. 7, Aa and Ac, top), respectively. Thus, a single wt Munc13-1 allele is sufficient to support PTP at calyx synapses. Consistent with previous studies (*28*, *40*), THFS increased the rate of mEPSCs (averaged over 2-s intervals) more strongly (wt/−: ~6.98-fold; Fig. 7, Ab and Ac, bottom, and table S3) than eEPSC amplitudes. THFS-induced changes in mEPSC amplitude estimates were very small (Fig. 7Ac, inset).

The PTP of eEPSC amplitudes was reduced by ~76% in HK/− synapses (*t*_0.5_ ≈ 73 s, *n* = 29; Fig. 7, Ba and Bc, top, and table S3), closely matching the reduction of PDBu-induced eEPSC potentiation. In contrast, the increase in mEPSC rates following THFS was slightly less affected (Fig. 7, Bb and Bc, bottom, and table S3). After PTP induction, mEPSC rates returned to control values more slowly in HK/− synapses (*t*_0.5_ ≈ 73 s) as compared to wt/− (*t*_0.5_ ≈ 43 s) synapses. To assess THFS-induced changes in STP in wt/− and HK/− synapses, 200-Hz eEPSC trains were recorded before and 15 s after THFS (Fig. 7, C and D). Because initial amplitudes and relative steady-state depression during 200-Hz trains increased strongly in wt/− synapses but changed only marginally in HK/− synapses following THFS, synapses of both genotypes exhibited remarkably similar strength and STP after PTP induction (Fig. 7Db).

### Bidirectional modulation of PTP by Munc13-1 C_2_B mutations that increase or decrease Ca^2+^-phospholipid binding

**Fig. 8.**
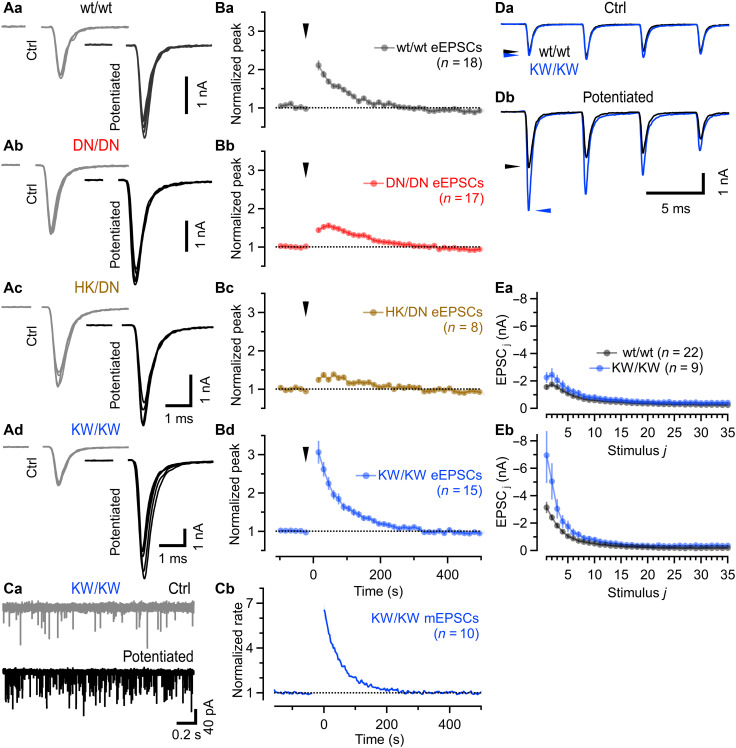
Mutations that reduce or enhance Ca^2+^-phospholipid binding of the Munc13-1 C_2_B domain attenuate or increase PTP of eEPSC amplitudes at calyx synapses. (**A**) Five consecutive eEPSCs recorded at 15-s intervals before (left, gray) and after (right, black) PTP induction (200 Hz, 8 s) in wt/wt (Aa), DN/DN (Ab), trans-heterozygous HK/DN (Ac), and KW/KW (Ad) synapses shown superimposed. (**B**) Mean time courses of eEPSC amplitudes for the same genotypes (Ba to Bd). Arrowheads indicate the THFS onset; *t* = 0 s marks the end of the tetanus. (**C**) Representative mEPSCs (Ca) before (gray, top) and after (black, bottom) PTP induction in KW/KW synapses. mEPSC rate potentiation was quantified from continuous recordings by calculating mean event rates over nonoverlapping 2-s bins (Cb). (**D**) Representative eEPSC trains elicited by 200-Hz (35 APs) stimulation and recorded before (Da) and 15 s after (Db) THFS in a wt/wt and in a KW/KW synapse shown superimposed. The first four eEPSCs in the trains are shown; arrowheads mark eEPSC_1_ amplitudes. (**E**) Mean eEPSC trains (200 Hz, 35 APs) before (Ea) and after (Eb) PTP induction in wt/wt and KW/KW synapses. Initial synaptic strength and STP are similar in wt/wt and KW/KW synapses under control conditions but differ strongly after PTP induction. Error bars [(Ba) to (Bd) and (E)] represent SEM. Numbers of synapses are given in parentheses for (Ba) to (Bd), (Cb), and (E).

Mutations that alter Ca^2+^-phospholipid binding to the Munc13-1 C_2_B domain perturb STP and recovery from depression in calyx of Held synapses (*59*). To evaluate the role of this domain in PTP, we measured PTP magnitude and time course in Munc13-1 C_2_B DN calyx synapses. Following THFS, calyx eEPSCs in wt/wt littermate mice were potentiated to a similar extent (*t*_0.5_ ≈ 60 s, *n* = 34; Fig. 8, Aa and Ba, and table S3) as in wt/− mice (Fig. 7, Aa and Ac, top). THFS-induced eEPSC potentiation was reduced in DN/DN synapses (Fig. 8, Ab and Bb, and table S3), although to a lesser extent than in HK/− synapses (Fig. 7, Ba and Bc, top) and decayed more slowly to half-maximum levels (*t*_0.5_ ≈ 143 s). Trans-heterozygous HK/DN synapses also exhibited reduced PTP (~1.37 ± 0.07-fold, *t*_0.5_ ≈ 86 s, *n* = 8; Fig. 8, Ac and Bc, and table S3), comparable in magnitude to that in HK/− synapses (Fig. 7, Ba and Bc, top). Thus, not only eEPSC recovery kinetics but also PTP was similarly impaired in HK/DN and HK/− synapses.

Because HK/− and DN/DN (*59*) calyx synapses exhibit increased synaptic strength even before THFS, their reduced PTP may reflect a ceiling effect on eEPSC amplitudes. To test this possibility, we (i) assessed the magnitude of PDBu-potentiation in DN/DN synapses (fig. S6) and (ii) measured PTP in Munc13-1^KW/KW^ (KW/KW) calyx synapses (Fig. 8, Ad and Bd), which express a Munc13-1 variant carrying a single point mutation that increases PIP_2_ binding without changing the Ca^2+^ affinity of the C_2_B domain (*75*).

The magnitude of PDBu-induced eEPSC potentiation in DN/DN synapses was, on average, nearly identical to that in wt/wt (fig. S6B) or wt/− (Fig. 2, B and E) calyx synapses. Thus, despite their strongly reduced PTP, synaptic strength of DN/DN synapses can, in principle, be increased at least twofold beyond resting values. KW/KW and wt/wt synapses exhibited comparable synaptic strength and STP during 200-Hz eEPSC trains before THFS (Fig. 8, Da and Ea) (*59*). Notably, PTP of eEPSCs was markedly enhanced in KW/KW synapses (*t*_0.5_ ≈ 41 s, *n* = 16; Fig. 8, Ad and Bd, and table S3), whereas THFS-induced potentiation of mEPSC rates was similar (Fig. 8C and table S3) to that observed in wt/wt and wt/− synapses. Following THFS, both the initial eEPSC and the extent of relative steady-state depression during stimulus trains were increased more strongly in KW/KW than in wt/wt synapses (Fig. 8, Db and Eb). The absolute eEPSC_1_ amplitudes following PTP induction were −5.87 ± 0.60 nA (*n* = 16) in KW/KW synapses but more than 45% smaller in DN/DN and HK/− synapses [−3.01 ± 0.25 nA (*n* = 23) and −2.56 ± 0.20 nA (*n* = 29), respectively].

Because PDBu still doubled eEPSC amplitudes in DN/DN synapses and PTP produced even larger eEPSCs in KW/KW synapses, the diminished PTP observed in HK/− and DN/DN synapses cannot be explained by a ceiling effect. Only ~22% of the normal PTP persisted in HK/− synapses, ~50% in DN/DN, and ~37% in HK/DN synapses (table S3). These findings argue against the HK C_1_ and DN C_2_B mutations acting on fully independent regulatory pathways. Instead, the HK C_1_ mutation not only abolishes DAG signaling but likely also disrupts the ability of Munc13-1 to sense THFS-induced changes in cytosolic Ca^2+^ via the C_2_B domain, consistent with impaired Ca^2+^-dependent eEPSC recovery in HK/− synapses.

## DISCUSSION

To explore the role of the Munc13-1 C_1_ domain for defining synaptic strength, shaping STP during stimulus trains, and inducing PTP, we studied calyx of Held synapses expressing Munc13-1 carrying the H567K C_1_ domain point mutation, which disables the domain and abolishes DAG binding. We circumvented perinatal lethality of Munc13-1^H567K/H567K^ mice (*18*) by selectively manipulating Munc13-1 expression in a subset of neurons.

### Levels and nanometer-scale organization of Munc13-1 at calyx AZ are unaffected by gene dosage

Despite ~19% reduced Munc13-1 expression levels in brains of juvenile heterozygous wt/− mice, wt/− and wt/wt calyx synapses were functionally indistinguishable. A single wt allele is therefore sufficient to maintain normal Munc13-1 function in wt/− calyx synapses, consistent with a previous study in cultured neurons (*5*) and likely reflecting both a saturating number of Munc13-1 copies in wt/wt synapses and a tightly regulated abundance of Munc13-1 at AZs via protein-protein interactions (*3*).

Analysis of Munc13-1 abundance at calyx AZs relative to that of BSN and of the Munc13-1 nanometer-scale organization provided no evidence for differences between wt/− and HK/− synapses, indicating that Munc13-1 localization to AZs is unaffected by the HK C_1_ mutation. Consistent with findings in cultured neurons (*76*), BSN nanoclusters at AZs were larger and positioned more centrally, whereas Munc13-1 nanoclusters were smaller, more numerous, and more peripherally located, with average distances of ~200 nm between Munc13-1 molecules and the center of the nearest BSN nanoclusters. Munc13-1 nanoclusters spatially overlap with RIM1/2, which is preferentially localized near SV release sites (*76*), whereas the central location of BSN nanoclusters within AZs is consistent with its proposed role as a structural scaffold. Although our estimates for the size and number of Munc13-1 nanoclusters per AZ closely match data obtained from cultured synapses (*12*, *76*), we cannot exclude the possibility that closely adjacent Munc13-1 nanoclusters were occasionally counted as a single cluster. Nonetheless, if Munc13-1 nanoclusters represent SV release sites (*12*, *13*), and given that calyces contain, on average, more than 500 AZs (*77*, *78*), this yields ~500 · 7.2 = 3600 SV release sites per calyx terminal, consistent with our model assumptions (table S2).

### The HK C_1_ mutation tilts the SV priming equilibrium in favor of fully primed TS SVs without altering TS SV fusion probability

Calyx of Held terminals express a large repertoire of presynaptic metabotropic transmitter receptor types (*79*), but there is no experimental evidence supporting DAG signaling through the Munc13-1 C_1_ domain via the G_q_/PLC-β pathway. The TrkB–PLC-γ pathway is the only known potential DAG source in calyx synapses, where genetic perturbation of BDNF expression or acute BDNF application affects glutamate release (*53*, *80*, *81*), and we show here that TrkB receptor agonist-mediated potentiation of glutamate release at calyx synapses depends on Munc13-1 C_1_ signaling.

Comparison of functional consequences of the HK C_1_ mutation in calyx synapses with previous works using *Caenorhabditis elegans* (*72*, *82*, *83*), *Drosophila melanogaster* (*84*–*86*), or cultured murine hippocampal neurons (*18*, *21*, *87*) is not straightforward owing to the differences in experimental conditions, synapse type, and genetic background. Transmitter release is enhanced in *C. elegans* expressing UNC13 C_1_ truncation variants (*72*, *82*), which is consistent with an inhibition of UNC13 priming activity by the C_1_ domain in the absence of DAG binding. Because a DAG binding–deficient HK UNC13 C_1_ point mutation increases transmitter release similarly, this mutation may render UNC13 partly disinhibited in addition to preventing DAG binding, thus functionally mimicking the C_1_ deletion (*82*).

In contrast to strongly enhanced initial eEPSCs and unchanged *FRP* size in HK/− calyx synapses, cultured HK/HK autaptic hippocampal neurons exhibit similar eEPSC amplitudes despite reduced *RRP* size, and faster SV pool depletion kinetics during hyperosmotic challenges (*18*, *21*). Considering a simple single-pool model of SV priming and fusion, these findings were interpreted as higher *p*_vr_, potentially reflecting increased SV fusogenicity (*18*, *21*, *24*). However, fusogenicity is primarily governed by proteins constituting the SV fusion machinery or regulating SV fusion kinetics, such as synaptotagmins, complexins, and SNAREs (*1*, *2*), which operate downstream of SV priming and control the final step of SV fusion upon presynaptic Ca^2+^ influx. Although Munc13-1 could regulate fusogenicity by determining the number of assembled SNARE complexes associated with docked SVs, our data clearly point to a role of Munc13-1–regulated SV priming in the potentiation of glutamate release in HK/− calyx synapses. Cultured autaptic hippocampal neurons expressing the disease variant C587F in the C_1_ domain of Munc13-1 exhibit largely unchanged *RRP* size as well as normal spontaneous and evoked transmission. Synapses carrying this C_1_ domain mutation are not potentiated by PDBu application (*88*), demonstrating that genetically induced loss of DAG binding is not necessarily associated with an increased release fraction (*F* = *p*_vr_ = eEPSC_1_/*RRP*).

In HK/− calyx synapses, the estimates for the release fraction *F* were ~1.8-fold higher compared to wt/− synapses, consistent with results obtained for cultured HK/HK autaptic neurons (*18*, *21*). Within the framework of the sequential two-step priming scheme, *F* is determined by the fraction of TS SVs multiplied by their fusion probability (*F* = *f*_TS_ · *p*_fusion_). Our detailed analysis of a large dataset of eEPSC trains by NTF decomposition (*65*) revealed that increased glutamate release in HK/− calyx synapses is primarily accounted for by a shift in the SV priming equilibrium toward a higher *f*_TS_, whereas *p*_fusion_ was largely unchanged. Thus, a higher abundance of TS SVs due to an increased rate constant of the LS→TS transition at rest (*k*_2,rest_) explains an increase in rapidly depletable transmitter release in HK/− calyx synapses. Because *p*_fusion_ and presynaptic Ca^2+^ influx were virtually unaltered in HK/− synapses, we find no evidence for increased SV fusogenicity.

Structural and molecular simulation data are consistent with our interpretation that HK/− synapses contain a higher fraction of fully primed TS SVs at rest but show impaired activity-dependent SV priming. Cryo–electron microscopy studies indicate that DAG and Ca^2+^/PIP_2_ binding to the Munc13 C_1_ and C_2_B domains promote a sequential transition from loosely to tightly docked, fusion-competent SVs (*73*). The H567K mutation favors the membrane-engaged, fully primed SV configuration under resting conditions (*89*) but disrupts DAG-dependent SV recruitment during activity, leading to rapid pool depletion and reduced steady-state release.

The frequency of spontaneously occurring mEPSCs was only slightly increased in HK/− calyx synapses. Previous studies reported either increased (*21*, *55*) or unchanged (*18*) rates of spontaneous release in cultured HK/HK synapses. Given the increased abundance of TS SVs in HK/− synapses, a higher mEPSC frequency might be expected, but it may be masked by a high variability of mEPSC rates among calyx synapses. On the other hand, it is not uncommon for genetic perturbations of synaptic proteins to affect spontaneous and AP-evoked release differentially (*90*, *91*), which may imply that SVs contributing to evoked or spontaneous release originate from different SV pools or that these genetic manipulations induce complex changes to the dose-response curve of transmitter release versus [Ca^2+^]_i_ (*92*).

### Munc13-1 is indispensable for potentiating calyx synapses by THFS

The assessment of PTP in calyx synapses expressing different Munc13-1 point mutations indicates that Munc13-1 is indispensable for this type of STP. The bidirectional changes in PTP induced by Munc13-1 C_2_B mutations that either decrease or increase Ca^2+^-phospholipid binding align with the established role of presynaptic residual [Ca^2+^]_i_ in PTP induction (*27*, *30*, *42*, *93*).

Pharmacological and genetic perturbations targeting PKCs strongly impair PTP at calyx synapses, implicating PKC signaling in PTP induction (*28*, *40*, *42*, *44*). The extent of reduction of PTP or PDBu-induced potentiation in HK/− synapses is very similar to that in PKCα/β DKO mice (*28*). This indicates that (i) either PKC and Munc13-1 converge on a common pathway, and activation of both is required for PTP induction and DAG/PDBu-induced eEPSC potentiation, so that impaired signaling via PKC or via Munc13-1 disrupts downstream signaling, or (ii) PKC acts upstream of Munc13-1, so that PKC activation enables and/or facilitates Munc13-1–mediated eEPSC potentiation. In support of the second mechanism, cryo–electron tomography studies revealed that PKC regulates SV interlinking (*94*), raising the possibility that PKC activation may enhance SV mobility. Preexposure to PDBu or high-frequency stimulation induces a permissive period during which PDBu-induced eEPSC potentiation is possible even in the presence of PKC blockers (*19*). Such preexposure may shift synapses into a state where signaling via Munc13-1 alone is sufficient to enhance transmitter release.

**Fig. 9.**
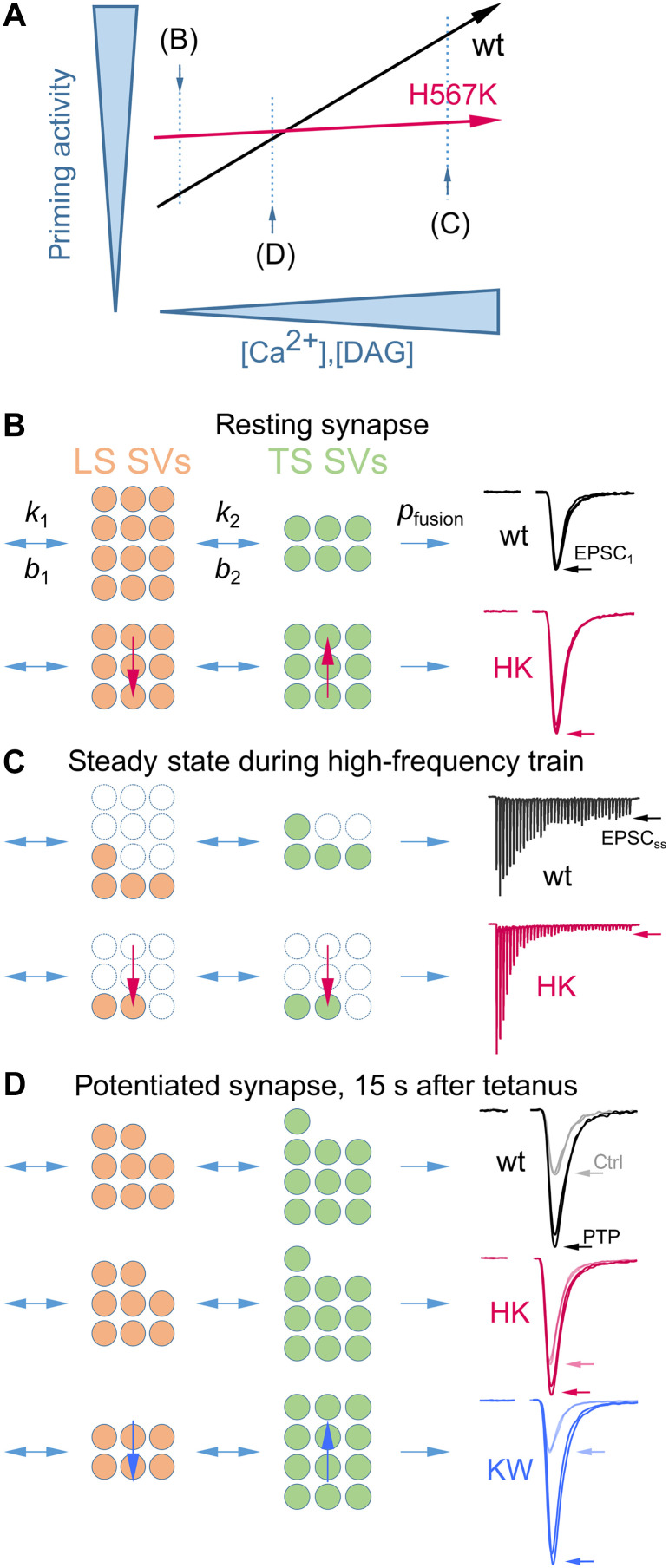
Dynamic Munc13-1–dependent SV priming regulates synaptic strength at rest, shapes STP during brief AP trains, and determines the magnitude of PTP. (**A**) In wt synapses, presynaptic Ca^2+^ and DAG signaling confer a broad dynamic range of Munc13-1–mediated SV priming. In HK/− synapses, this range is reduced due to elevated SV priming activity at rest and a smaller activity-dependent increase during AP firing. (**B** to **D**) Schematics of mutation-induced changes (vertical arrows) in relative abundances of LS versus TS SVs (number of light red versus light green circles) for three activity contexts [letters in (A)]. (B) In resting wt terminals (top), Munc13-1 activity is partially inhibited by the C_1_ domain. Relief of this inhibition in resting HK/− terminals (bottom) increases the abundance of fully primed fusion-competent TS SVs among all *FRP* SVs [*f*_TS_ = TS/(LS + TS)], thereby enhancing basal synaptic strength. (C) During high-frequency AP trains, SV priming is up-regulated, counteracting SV depletion and sustaining transmission (top). This Ca^2+^-dependent SV priming augmentation is impaired in HK/− synapses (bottom), reducing the availability of fusion-competent TS SVs and thus reducing steady-state glutamate release. Empty circles represent the depleted *FRP* fraction. (D) During and after tetanic stimulation in wt synapses, Ca^2+^ and DAG signaling via Munc13-1 C_1_ and C_2_B domains increase *f*_TS_, enabling PTP. eEPSC potentiation relative to basal synaptic strength is reduced in HK/− (impaired DAG binding) and DN/DN (impaired Ca^2+^-phospholipid binding) synapses but enhanced in KW/KW synapses (increased Ca^2+^-phospholipid binding). The total *FRP* size (LS + TS) is assumed to be nearly unaltered in (B) and (D).

Following THFS, the increase in mEPSC rate exceeded the potentiation of eEPSC amplitude, reflecting, in part, the different [Ca^2+^] levels mediating spontaneous and evoked release (*51*). About 15 s after THFS, eEPSC amplitudes were potentiated about twofold, corresponding to an elevation of global [Ca^2+^]_i_ by ~80 nM above its resting level of ~50 nM (*27*, *95*). Assuming a slope of ~1.73 for the log(mEPSC rate) versus log([Ca^2+^]) relationship (*27*), this predicts an increase in mEPSC frequency of ${\left(130/50\right)}^{1.73}\approx 5.22$-fold, consistent with experimental observations (*28*, *40*). In contrast, for AP-evoked release, using a slope of ~4.2 for the log(peak release rate) versus log([Ca^2+^]) relationship (*51*) and assuming an ~80 nM increase in the peak of the local [Ca^2+^]_i_ from ~56.00 μM (*96*) to ~56.08 μM, the predicted change is only ${\left(56.08/56\right)}^{4.2}\approx 1.006$-fold. This small increase is far below the ~2.0-fold enhancement of eEPSC amplitude observed experimentally, indicating that additional mechanisms must account for most of the PTP of evoked release. A tilted LS-TS priming equilibrium can readily provide such an explanation (Fig. 9).

### Interdependence of C_1_ and C_2_B signaling for regulating Munc13-1–dependent SV priming

Similar to cultured HK/HK autaptic neurons (*18*), HK/− calyx synapses exhibit stronger steady-state depression and slower eEPSC recovery following high-frequency stimulation. Activity-dependent acceleration of SV pool replenishment is enabled through Munc13-1 CaM binding and Ca^2+^-phospholipid binding to the C_2_B domain (*59*, *71*) and is also affected by the loss of presynaptic proteins (*62*, *97*–*101*) that are directly or indirectly regulated by Ca^2+^. Although the Munc13-1 C_1_ domain can bind DAG independently of Ca^2+^ (*14*), the HK C_1_ mutation abolished the fast eEPSC recovery component. This implies that either rapid changes in intracellular concentrations of membrane-bound DAG, on a timescale of tens of milliseconds, contribute to regulating pool replenishment kinetics or, more likely, that the HK C_1_ mutation interferes with intramolecular interactions of the C_1_ domain with the closely adjacent C_2_B domain (*21*, *72*), which impairs C_2_B Ca^2+^ sensing. C_1_ and C_2_B domains are in close proximity and cooperatively mediate DAG-dependent and Ca^2+^-dependent membrane binding (*74*, *102*), indicating a level of interdependence that could be perturbed by the HK C_1_ domain mutation.

Impaired eEPSC recovery kinetics and reduced PTP in trans-heterozygous HK/DN calyx synapses indicate that the function of Munc13-1 is preserved only if the C_1_ and C_2_B domains are both intact in the same molecule. Furthermore, autaptic neurons expressing a Munc13-1 variant with a R750E and K752E double mutation, which disrupts the salt bridges between the C_1_ and C_2_B domains, exhibit reduced PDBu-induced potentiation and enhanced synaptic depression during high-frequency stimulation (*74*). In *C. elegans*, the C_1_ domain stabilizes an inhibitory function of C_2_B, and activating the C_1_ domain by phorbol esters relieves this inhibition. Although deletion of either domain alone increases transmitter release, their combined loss severely disrupts SV priming and fusion, likely due to impaired Munc13-1 localization at the AZ (*72*). DAG binding to the C_1_ domain was proposed to position SVs at an ~12-nm distance from the plasma membrane, whereas subsequent Ca^2+^ binding to the C_2_B domain further repositions SVs closer to the plasma membrane, promoting SNARE assembly (*75*). Such a sequential SV priming scheme implies that DAG-dependent activation of the C_1_ domain occurs upstream of Ca^2+^-induced C_2_B activation, which is consistent with the intact PDBu-induced eEPSC potentiation in DN/DN calyx synapses and the impaired activity-dependent acceleration of SV priming and reduced PTP in HK/− synapses.

### The HK Munc13-1 C_1_ mutation—Gain-of-function or loss-of-function?

The ~1.8-fold increase in *F* in HK/− calyx synapses is consistent with the higher *p*_vr_ estimates obtained in cultured HK/HK autaptic neurons (*18*, *21*), which led to the conclusion that the Munc13-1 HK mutation is a gain-of-function mutation that mimics the PDBu-activated state of Munc13-1 (*21*). However, major differences between HK/− calyx synapses and PDBu-treated wt calyx synapses exist: PDBu application enhances steady-state release, accelerates recovery from depression (*22*, *23*), and increases spontaneous mEPSC rates in wt calyx synapses, whereas HK/− calyx synapses show reduced steady-state release, slower recovery, and unchanged spontaneous glutamate release (fig. S5). Previous studies found that PDBu application either increases *RRP* size (*22*, *23*, *103*, *104*) or does not affect it (*21*, *24*). Cultured HK/HK autaptic neurons, on the other hand, exhibit a 44% smaller *RRP* (*18*, *21*), whereas the *FRP* size in HK/− calyx synapses, representing the sum of LS and TS subpools, was unaltered (Fig. 4E). Considering these differences, the notion that the Munc13-1 HK C_1_ mutation mimics the PDBu-bound state of Munc13-1 seems unwarranted. Our kinetic modeling indicates a “gain-of-function” with respect to the parameters defining the LS→TS transition at rest and during activity (*k*_2,rest_ and σ_2_), and a “loss-of-function” with respect to the parameter defining the ES→LS transition during activity (σ_1_). Collectively, these parameter changes cause higher initial synaptic strength and increased steady-state release during low-frequency stimulation but reduced steady-state release and slower eEPSC recovery during and following high-frequency stimulation.

### Munc13-1–dependent modulation of synaptic strength in resting and active calyx synapses

Our data support the following model for Munc13-1–mediated regulation of synaptic strength and plasticity: DAG and Ca^2+^ signaling via the Munc13-1 C_1_ and C_2_B domains dynamically modulate SV priming activity. In wt calyx synapses, Munc13-1–mediated SV priming operates over a broad dynamic range, increasing with presynaptic cytosolic Ca^2+^ and/or DAG concentrations (Fig. 9A). At rest, the C_1_ domain maintains Munc13-1 in an autoinhibited state (*21*, *72*, *82*, *83*), which limits SV priming. DAG binding or the HK C_1_ mutation, which disrupts C_1_ domain structure, relieves the autoinhibition and enhances synaptic strength due to an increased abundance of TS SVs (Fig. 9B). During high-frequency activity, elevated [Ca^2+^]_i_ is essential to sustain SV replenishment and glutamate release (*66*). Activity-dependent acceleration of SV priming requires intact C_1_ and C_2_B domains. Disruption of either domain leads to slower SV pool replenishment, enhanced pool depletion, and increased synaptic depression during continuous activity in HK/− and DN/DN calyx synapses. This is reflected by reduced occupancy of both TS and LS vesicle states in HK synapses (Fig. 9C). Tetanic stimulation of wt synapses transiently increases the abundance of TS SVs through DAG and Ca^2+^ signaling via the Munc13-1 C_1_ and C_2_B domains (Fig. 9D). This THFS-induced shift in LS-TS balance is impaired in HK/− and DN/DN calyx synapses, resulting in diminished PTP. However, reduced PTP in these synapses is not caused by a ceiling effect as PDBu still potentiates eEPSCs in DN/DN synapses, and eEPSCs measured after PTP induction in KW/KW synapses are substantially larger than initial eEPSCs in HK/− and DN/DN synapses.

## MATERIALS AND METHODS

### Mouse maintenance

Mouse line generation and experimentation were approved by the authorities of the local government (Lower Saxony State Office for Consumer Protection and Food Safety, LAVES; permit 33.19-42502-04-15/1817). Animals were maintained in accordance with European Union Directive 63/2010/EU and ETS. Animal health was controlled daily by caretakers and by a veterinarian, and a quarterly health monitoring was done according to FELASA recommendations. Mice were kept in individually ventilated cages, under specific pathogen–free conditions, at 21° ± 1°C, 55% relative humidity, and 12-hour/12-hour light/dark cycle. Food and tap water, as well as bedding and nesting material, were provided ad libitum, and cages were changed once a week. Mice were routinely genotyped by polymerase chain reaction (PCR). We did not observe large deviations from the expected Mendelian ratios among offspring and neither the brainstem-specific HK/− mice nor their wt/− littermate mice exhibited any phenotypic abnormalities.

### Mouse generation

The generation of Munc13-1^H567K^ mutant mice has been previously reported (*49*). Homozygous Munc13-1^H567K/ H567K^ mice die shortly after birth. We circumvented perinatal lethality by introducing a Munc13-1^−^ allele in a subset of neurons, including globular bushy cells of the AVCN. This approach enabled a comparison of functional properties of heterozygous Munc13-1^HK/−^ and Munc13-1^wt/−^ calyx synapses. Heterozygous Munc13-1^H567K/wt^ mice were crossbred with the Krox20 cre driver line (Krox20^cre/wt^) to generate Munc13-1^H567K/wt^ Krox20^cre/wt^ mice. Krox-20 is a transcription regulatory factor that is expressed in the brainstem, including globular bushy cells, and cerebellar neurons starting at E17 (*47*, *48*, *105*). The Krox20^cre/wt^ mouse line has previously been used to generate brainstem-specific RIM1,2 double knockout mice (*48*). Thereafter, Munc13-1^H567K/wt^ Krox20^cre/wt^ mice were crossbred with homozygously floxed Munc13-1 (Munc13-1^fl/fl^) mice to produce brainstem-specific Munc13-1^H567K/−^, Krox20^cre/wt^ mice, as well as Munc13-1^wt/−^, Krox20^cre/wt^ littermates (Fig. 1A). Body weights of Munc13-1^H567K/−^ and Munc13-1^wt/−^ mice were indistinguishable (fig. S1E). Munc13-1 conditional knockout mice (Unc13a^tm1.2Sud^) where exon 18 is targeted for deletion were generated by homologous recombination and validated via long-range PCR and sequencing reactions. For routine genotyping, the following primer sequences were used: GGGTAGCTGCAGGATTTATTGTAT (forward) and TGTGTCCAGTTTCAGAGGTC (reverse).

To confirm the loss of Munc13-1 expression after Cre-Lox recombination of the conditional Munc13-1 allele, Munc13-1^wt/fl^ mice were crossbred with EIIa-cre mice (*106*), which express Cre recombinase in nearly all tissue of the early embryo, to generate Munc13-1^wt/−^ mice. Munc13-1^wt/−^ mice were subsequently bred with Munc13-2 ^wt/−^ mice to produce Munc13-1^−/−^, Munc13-2^−/−^ mouse embryos. Western blot analysis revealed the absence of Munc13-1 protein in E18 brains of Munc13-1^−/−^ mice without compensatory up-regulation of Munc13-2, whereas Munc13-1 levels were only slightly reduced in E18 and P21 brains of Munc13-1^wt/−^ mice, respectively (fig. S1, B and C).

### Western blotting

Whole brains of E18 embryos or cortices of P21 pups were homogenized and centrifuged for 10 min at 1000*g* to collect the post–nuclear supernatant. Protein samples containing 10 to 20 μg of total protein were separated on 4 to 12% gradient Bis-Tris polyacrylamide gels (Invitrogen) and blotted onto nitrocellulose membranes. Primary antibodies used were rabbit-anti-Munc13-1 (Synaptic Systems, 126103) at 1:2000 and guinea pig-anti-Munc13-2 (Synaptic Systems, 126205) at 1:400. Mouse-anti-β-Tubulin (Merck, TUB 2.1 T4026) at 1:20,000 was used as a loading control. Secondary antibodies used were horseradish peroxidase (HRP) coupled goat-anti-rabbit (Jackson ImmunoResearch, 111-035-144), goat-anti-guinea pig (Jackson ImmunoResearch, 106-035-003), and goat anti-mouse (Jackson ImmunoResearch, 115-035-146), each at 1:10,000. Enhanced chemiluminescence (ECL) (Amersham, RPN3004) was documented using an Intas ECL Chemostar Plus Imager (Intas).

### HEK293FT cell culture and transfection

Human embryonic kidney (HEK) 293FT cells were cultured in 10-cm dishes at 37°C with 5% CO_2_ in growth medium consisting of Dulbecco’s modified Eagle’s medium (DMEM) supplemented with GlutaMAX (Gibco), penicillin-streptomycin (100 U/ml), and 10% fetal bovine serum (FBS; Gibco). At 80 to 90% confluency, cells were washed with phosphate-buffered saline (PBS) and treated with 0.05% trypsin/EDTA (Thermo Fisher Scientific) at 37°C for 2 min to detach them. Detached cells were resuspended in fresh medium and replated at the desired density. Once cultures reached 50 to 60% confluency, cells were transfected with either pEGFP-N1-Munc13-1^wt^, pEGFP-N1-Munc13-1^HK^, or an empty pEGFP-N1 vector, along with pCMV-RIM1-mCherry using Lipofectamine 2000 and according to the manufacturer’s protocol.

### Coimmunoprecipitation

Transfected HEK cells were washed once with 10 ml of Dulbecco’s modified phosphate buffered saline and harvested in 1 ml ice-cold IP buffer containing 0.15 M NaCl, 0.05 M tris-HCl (pH 7.4), 1 mM CaCl_2_, 1% NP-40, aprotinin (1 μg/ml; Rapid Test Methods Ltd.), leupeptine (0.5 μg/ml; Peptide Institute Inc.), and phenylmethylsulfonyl fluoride (PMSF; 17.4 μg/ml; Sigma-Aldrich). The cell lysates were incubated with gentle agitation at 4°C for 10 min with 0.5 μl of Benzonase Nuclease (Merck) and centrifuged at 100,000*g* at 4°C for 30 min, and the protein concentration of the soluble supernatant was measured using the Pierce BCA Protein-Assay (Thermo Fisher Scientific). Three milligrams of cell lysates was mixed with 30 μl of GFP-Trap magnetic Agarose beads (ChromoTek) after the beads had been equilibrated in IP buffer through three short washes. After 1.5-hour incubation with gentle agitation at 4°C, the beads were washed five times with washing buffer (IP buffer, but with 0.1% NP-40) and eluted in 1× Laemmli buffer by boiling the beads at 95°C for 10 min. Western blot samples were loaded on a NuPAGE Novex 12% Bis-Tris Protein Gel (Thermo Fisher Scientific), and proteins were separated and transferred onto a nitrocellulose membrane. The membrane was washed with ddH_2_O, and proteins were stained with the MemCode Reversible Protein Stain Kit (Thermo Fisher Scientific) to control for loading accuracy. The protein stain was removed, and blocking buffer was added for 30 min [tris-buffered saline (TBS): 0.1 M NaCl, 10 mM Tris, pH 7.4 supplemented with 0.1% Tween 20 and 5% milk powder]. Next, a primary antibody [anti-GFP (Enzo, ADI-SAB-500-E, 1:2000) or anti-RFP (Synaptic Systems, 390004, 1:1000)] was diluted in blocking buffer and added to the membrane for 2 hours. After three washes with blocking buffer, the membrane was incubated for 1 hour in HRP-coupled secondary antibodies [Goat Anti-Mouse IgG (H+L), Jackson 115-035-146 or Goat Anti-Guinea Pig IgG (H+L), Jackson 106-035-003] diluted 1:10,000 in blocking buffer. Last, the membrane was washed three times with TBST buffer (0.1 M NaCl, 10 mM Tris, and 0.1% Tween 20, pH 7.4) and once with 1x TBS buffer. The chemiluminescence signal was developed with Amersham ECL Western Blotting Detection Reagents (GE Healthcare Life Sciences) and captured using a Gel iX Imager (INTAS Science Imaging Instruments).

### Immunohistochemistry

Immunostaining was conducted on coronal brainstem sections from P18 mice, targeting Munc13-1, bassoon, and vGluT1. Mouse brains were rapidly frozen in isopentane at −35°C. Twelve-micrometer-thick coronal sections of the medial nucleus of the trapezoid body (MNTB) were cut in cryostat at −17°C and mounted on superfrost slides. Sections containing HK/−, wt/−, and wt/wt calyx terminals were processed together. They were air-dried for 15 min and immersion fixed using ice-cold 4% paraformaldehyde (PFA) [in 0.1 M phosphate buffer (PB), pH 7.4, 6 min, room temperature (RT)]. STORM samples were additionally quenched with 0.1% NaBH_4_ for 7 min before PFA fixation. Sections were washed with 0.1 M PB, and nonspecific binding was blocked (90 min, RT). For confocal imaging, the blocking solution contained 5% normal goat serum (NGS), 0.1% cold water fish skin gelatin, and 0.5% Triton X-100 in 0.1 M PB (pH 7.4). For STORM imaging, sections were blocked in 10% NGS and 0.5% Triton X-100 in 0.1 M PB (pH 7.4). They were then incubated overnight at 4°C with the following primary antibodies: rabbit polyclonal anti-Munc13-1 (SySy 126 103, 1:400), mouse monoclonal anti-bassoon (Enzo Life Sciences SAP7F407, 1:400), and guinea pig polyclonal anti-vGluT1 (SySy 135 304, 1:1400), which were diluted in incubation buffer (pH 7.4) containing 0.1 M PB, 3% NGS, 0.1% cold water fish skin gelatin, and 0.3% Triton X-100 for confocal samples or 5% NGS and 0.5% Triton X-100 for STORM samples. After extensively washing in PB, sections were incubated with the following fluorescent secondary antibodies in the dark for 2 hours at RT: Alexa Fluor 488–coupled goat anti-rabbit, Alexa Fluor 555–coupled goat anti-mouse, and Alexa Fluor 633–coupled goat anti-guinea pig (Invitrogen, 1:1000), which were diluted in incubation buffer. After washing, coverslips were mounted using Aqua-PolyMount for the confocal samples. STORM samples were postfixed in 4% PFA for 10 min, rinsed with 0.1 M PB, and stored in 0.1 M PB until imaging.

### Confocal microscopy and image analysis

Images were acquired using a laser-scanning confocal microscope (Leica TCS SP8; Leica Microsystems) equipped with a 63× [1.5 numerical aperture (NA)] oil immersion objective. Single-plane micrographs and *Z*-stacks were captured in sequential scanning mode with the same pixel size and pinhole settings for all three channels (vGluT1, bassoon, and Munc13-1). Laser power and gain settings were adjusted such that collected signals fell within the linear range of detection. Subsequently, confocal images were subjected to deconvolution using Leica Lightning Deconvolution software (Leica Microsystems).

Image analysis was performed using Fiji (ImageJ). To quantify a Munc13-1/BSN intensity ratios (Fig. 3Ba), ROIs were defined around MNTB cell bodies where vGluT1 expression was detected. The mean ratio of Munc13-1 to BSN signal intensity was quantified within ROIs across all images in the *Z*-stack. For each ROI, the two images within the *Z*-stack exhibiting the highest vGluT1 signal intensity were identified. A total of 12 different ROIs were analyzed, which were derived from seven to nine different calyx terminals for each of the three genotypes.

To assess vGluT1 hotspot enrichment (Fig. 3Bb), a polygon ROI was drawn for each calyx in Fiji using the vGluT1 channel, and a thin band immediately outside the ROI was used to estimate the local background (with mean μ_bg_ and SD σ_bg_). A duplicate image was converted to 32-bit and *z*-normalized [*z* = (*I* − μ_bg_)/σ_bg_] to express the vGluT1 intensity (*I*) in SDs above local background (*z*-image). Subsequently, the median intensity (*z*50) of the ROI was obtained, and the 90th percentile (*z*90) was determined as the intensity threshold that yielded an area fraction equivalent to 10% of the original size of the ROI of the *z*-image. The vGluT1 hotspot enrichment was defined as *z*90/*z*50.

### 3D stochastic optical reconstruction microscopy

Super-resolution images were recorded with a Vutara VXL (Bruker Nanosurfaces) commercial microscope based on single-molecule localization biplane technology (*107*, *108*). Samples were imaged in oxygen scavenging buffer containing thiols. STORM buffer: Buffer A [50 mM tris-HCl (pH 8.0) and 10 mM NaCl] + 10% (w/v) glucose, glucose oxidase [168 units (U)], bovine catalase (1400 U), 20 mM cysteamine, and beta-mercaptoethanol (1%). Imaging was performed for no more than 2 hours after STORM buffer was mixed for optimal oxygen scavenging. Samples were imaged with a 640-nm excitation power of ~500 mW, 555-nm excitation power of ~1000 mW, and 488-nm excitation power of ~300 mW. Images were acquired using a 60×/1.3 NA silicon immersion objective and a Hamamatsu Orca Fusion BT sCMOS camera (Hamamatsu Photonics) at 50 Hz. A total of 8500 frames per fluorophore were recorded, except for vGluT1 (2500 frames). Frames were analyzed by the Vutara SRX software (version 7.0.0rc39; Bruker Nanosurfaces). Frames that contained nonblinking fluorophores were not analyzed. Single molecules were identified by their brightness frame-by-frame after removing the background. Identified molecules were localized in three dimensions by fitting the raw data in a 12 × 12-pixel ROI centered around each particle in each plane with a three-dimensional (3D) model function that was obtained from recorded bead data sets. Fit results were filtered by a density-based denoising algorithm to remove isolated particles. The experimentally achieved image resolution of 20 nm laterally (*x*,*y*) was determined by Fourier ring correlation. Localizations were rendered as 25-nm particles.

### 3D STORM data analysis

Data collection was restricted to calyx AZs that were in focus, en face orientation, and positive for vGluT1. NNDs were calculated using Vutara SRX software (version 7.0.0rc39; Bruker Nanosurfaces). Particle clustering was performed using the DBScan cluster algorithm (*109*). Cluster statistics were taken from the output of DBScan. For measurements of cluster size, the radius of gyration (*r_g_*) was taken. The radius of gyration is a measure for the distribution of molecules within a nanocluster and represents the root mean square distance of *N* molecules from their center of mass $\left({\vec{r}}_{m}\right)$ within a given nanocluster ${r_{g}}^{2}=\frac{1}{N}\cdot \sum_{i=1}^{N}r_{i}$. The center of mass is defined as ${\vec{r}}_{m}=\sum_{i=1}^{N}{\vec{r}}_{i}$, where *r* is a vector in 3D Euclidean space with coordinates (*x*,*y*,*z*).

### Acute slice preparation

Acute brainstem slices of P15 to P17 mice of either sex were prepared as previously described (*59*). After decapitation, the whole brain was immediately immersed into ice-cold low-Ca^2+^ artificial cerebrospinal fluid (ACSF) containing 125 mM NaCl, 2.5 mM KCl, 0.1 mM CaCl_2_, 3 mM MgCl_2_, 25 mM glucose, 25 mM NaHCO_3_, 1.25 mM NaH_2_PO_4_, 0.4 mM ascorbic acid, 3 mM myoinositol, and 2 mM Na-pyruvate (pH 7.4, bubbled with 95% O_2_ and 5% CO_2_). The brainstem was glued onto the stage of a VT1000S vibratome (Leica Microsystems), and 190-μm-thick coronal slices containing the medial nucleus of the trapezoid body (MNTB) were cut. Slices were incubated for 50 min at 35°C in a chamber containing normal ACSF (identical to low-Ca^2+^ ACSF, except that 3 mM MgCl_2_ and 0.1 mM CaCl_2_ were replaced with 1.3 mM MgCl_2_ and 1.7 mM CaCl_2_). Slices were kept at RT (21° to 24°C) and used for recordings for up to 5 hours after recovery.

### Acute slice electrophysiology

Whole-cell patch-clamp recordings were made from calyx of Held terminals and PNs of the MNTB at RT using an EPC-10 amplifier controlled by Pulse or Patchmaster software (HEKA Electronics). Patch pipettes for postsynaptic recordings were pulled from borosilicate glass (Science Products) and had an open-tip resistance of 2.5 to 4 MΩ. They were filled with filled with a Cs-gluconate–based solution containing 100 mM Cs-gluconate, 30 mM TEA-Cl, 30 mM CsCl, 10 mM Hepes, 5 mM EGTA, 5 mM Na_2_-phosphocreatine, 4 mM ATP-Mg, and 0.3 mM GTP, pH 7.2 with CsOH. During experiments, slices were continuously perfused with normal ACSF solution containing 1.3 mM MgCl_2_ and 1.7 mM CaCl_2_ and supplemented with 5 μM strychnine to block glycinergic inputs. Cells were visualized by infrared–differential interference contrast microscopy through a 40× water-immersion objective using an upright BX51WI microscope (Olympus). All experiments were performed at RT.

A bipolar stimulation electrode was used to evoke presynaptic APs (stimulus intensity ≤ 20 V, 100-μs duration). Series resistance (*R*_s_) was ≤8 MΩ and compensated ≥82%. The holding potential (*V*_h_) and leak current were −70 mV and ≤300 pA, respectively. Sampling interval and low-pass filter settings were 20 μs and 5.0 kHz, respectively. To reduce eEPSC amplitudes for improved voltage-clamp and to attenuate postsynaptic AMPAR saturation and AMPAR desensitization, all experiments were performed in the continuous presence of the low-affinity GluR antagonists kyn (1 mM; Figs. 1, 2, 5, 7, and 8) or γ-DGG (2 mM; Fig. 4), unless explicitly stated otherwise. Furthermore, voltage-clamp errors caused by the remaining uncompensated *R*_s_ were corrected offline by a software routine courtesy provided by E.N. All offline analysis of electrophysiological data and all numerical simulations were performed using Igor Pro (WaveMetrics).

Presynaptic voltage-clamp recordings were performed using patch pipettes with an open-tip resistance of 3 to 4.5 MΩ. Series resistance was ≤15 MΩ and compensated 60 to 65%. For measuring *I*_Ca(V)_ and membrane capacitance (*C*_m_), pipettes were filled with a Cs-gluconate–based solution containing 100 mM Cs-gluconate, 30 mM TEA-Cl, 30 mM CsCl, 10 mM Hepes, 0.05 mM EGTA, 5 mM Na_2_-phosphocreatine, 4 mM ATP-Mg, and 0.3 mM GTP, pH 7.3 with CsOH. The bath solution was supplemented with 1 μM tetrodotoxin (TTX), 1 mM 4-Aminopyridine (4-AP), and 40 mM TEA-Cl to suppress voltage-gated sodium and potassium currents. The *V*_h_ and leak current were −80 mV and ≤100 pA, respectively. Changes in presynaptic membrane capacitance (∆*C*_m_) were measured using the Sine+DC technique with a software lock-in amplifier (HEKA Electronics) by adding a 1-kHz sine wave voltage command (±35-mV peak-to-peak amplitude) to *V*_h_ = −80 mV. Calyx terminals were visualized by oblique illumination (Dodt gradient contrast) through a 60× water-immersion objective using an upright BX51WI microscope (Olympus). All experiments were performed at RT.

### Neuronal mass culture and electrophysiology

Glass coverslips were coated with poly-d-lysine before cell plating. The brain of E18 embryos was rapidly extracted and placed in ice-cold Hanks’ balanced salt solution (HBSS) supplemented with 1 mM kyn. Hippocampi were dissected out and transferred to 0.5 ml of prewarmed papain solution (25 U/ml) and incubated at 37°C with shaking (450 rpm) for 45 min. Stop solution was added, followed by incubation at 37°C with shaking for an additional 15 min. The supernatant was carefully removed to avoid tissue loss. After digestion, the tissue was resuspended in 300 μl of plating medium. The plating medium consisted of 49 ml of Neurobasal-Plus (Gibco Thermo Fisher Scientific), 1 ml of B27 Supplement, 0.125 ml of GlutaMAX (200 mM), 0.1 ml of penicillin-streptomycin, and 0.1 ml of glutamate (12.5 mM) and FBS. Cells were triturated several times, and the supernatant was collected through a 40- to 100-μm cell strainer every time into a 50-ml tube. The cell density was determined using a cell counter. Cells were plated at a density of 250,000 per well. The plated cultures were maintained under standard conditions at 37°C in a humidified incubator with 5% CO_2_. A 50% medium was replaced with fresh Neurobasal-Plus medium containing B27, GlutaMAX, and penicillin-streptomycin but lacking FBS and glutamate at day in vitro (DIV) 1 (24 hours postplating). From DIV 2 onward, 50% of the culture medium was replaced with BrainPhys (STEMCELL Technologies) neuronal medium and this process was repeated every 3 to 4 days.

Whole-cell voltage-clamp recordings (*V*_h_ = −70 mV) were acquired at RT using an EPC-10 amplifier and Patchmaster software (HEKA Electronics). The standard extracellular solution contained 140 mM NaCl, 2.4 mM KCl, 10 mM Hepes, 10 mM glucose, 2 mM CaCl_2_, and 4 mM MgCl_2_ (305 to 307 mosmol/liter, pH 7.4) and was supplemented with 20 μM bicuculline. The intracellular solution used for experiments on cultured neurons was identical to the one used in patch-clamp recordings of MNTB PNs. For estimating current responses elicited by hyperosmotic stimulation, the standard extracellular solution was supplemented with 500 mM sucrose and 1 μM TTX. A custom-made fast-flow perfusion system, composed of glass capillaries controlled by a stepper motor, enabled rapid and complete exchange of extracellular solutions surrounding the recorded neuron. The sucrose response were measured at DIV 8. mEPSC recordings were performed at DIV 14 to 17.

### Statistical analysis

Data were analyzed using WaveMetrics Igor Pro and R software, and are presented as means ± SEM. If not stated otherwise, a two-tailed Welch-Satterthwaite *t* test was used to test for statistical significance of the differences between two sample means. Differences between multiple samples were evaluated using Tukey’s multiple comparisons of means. The statistical details of individual experiments including the *n* values are given in the figure legends and supplemental tables.

## Supplementary Materials

### This PDF file includes:

Supplementary Text Figs. S1 to S7 Tables S1 to S3 References
