# Supplementary material for: Munc13-1 couples DAG and Ca2+ signaling to dynamic vesicle priming, synaptic short-term plasticity, and posttetanic potentiation
Source: Sci Adv. 2026 Feb 13;12(7):eaea0449. doi: 10.1126/sciadv.aea0449 (PMC12904202; doi:10.1126/sciadv.aea0449)
Supplement: Supplementary file 1 — Supplementary Text Figs. S1 to S7 Tables S1 to S3 References [file sciadv.aea0449_sm.pdf]

Science Advances
AAAS

Supplementary Materials for

Munc13-1 couples DAG and Ca^{2+} signaling to dynamic vesicle priming, synaptic short-term plasticity, and posttetanic potentiation

Mrinalini Ranjan *et al.*

Corresponding author: Noa Lipstein, lipstein@fmp-berlin.de; Nils Brose, brose@mpinat.mpg.de;
Holger Taschenberger, taschenberger@mpinat.mpg.de

Sci. Adv. **12**, eaea0449 (2026)
DOI: 10.1126/sciadv.aea0449

This PDF file includes:

Supplementary Text
Figs. S1 to S7
Tables S1 to S3
References

Supplementary Text

Estimating the FRP from high-frequency eEPSC trains

At calyx of Held synapses, the SV pool that can be released within ~50 ms in response to strong and prolonged presynaptic $[Ca^{2+}]_i$ elevations induced by presynaptic Ca^{2+} uncaging or direct presynaptic depolarizations can be subdivided into two main SV subpools – 'fast releasing' (FRP) and 'slowly releasing' (SRP) SVs (*52*). SRP SVs only marginally contribute to AP-evoked release (*110*), such that SV pool estimates derived from AP-evoked eEPSCs primarily represent the FRP. Estimates for the FRP were obtained from cumulative eEPSCs in response to high-frequency trains (50, 100, 200 and 333 Hz). Cumulative eEPSC amplitudes were corrected for SV recruitment by fitting a line to the final five eEPSCs representing the steady state and back-extrapolating that line to $eEPSC_1$ (SMN method) (*111*). Such approach rests on the assumptions that (*i*) all steady-state release is balanced by SV replenishment which occurs at constant rate throughout a stimulus train, and that (*ii*) the FRP is fully emptied after each AP under steady-state conditions. Since neither of the two conditions is fully met, the back-extrapolation approach is expected to underestimate the actual FRP size (*111*). We confirm this by observing that FRP estimates obtained at 50, 100 and 200 Hz stimulation were consistently lower than those obtained at 333 Hz stimulation. We therefore refer to such estimates as apparent pool sizes (FRP'). When plotting $1/FRP'$ for 50, 100 and 200 Hz as a function of $1/f_{stim}$, where f_{stim} is the respective stimulation frequency, we notice that this relationship is nearly linear, and we can therefore obtain a corrected FRP estimated for the case $1/f_{stim} = 0$ (corresponding to infinite f_{stim}) by linear regression as the inverse of the intercept of such regression line (corrected SMN method; Fig. 4E) (*23*, *25*, *59*).

Sequential two-step LS-TS kinetic scheme for SV priming and fusion

Time courses of AP-evoked synchronous release during stimulus trains were simulated using a kinetic scheme previously described (fig. S7) (*25*). This kinetic scheme is based on the following assumptions:

1. docking/priming of SVs occurs at a single type of release site and the total number (N_{total}) of functionally identical release sites is fixed,

2. SVs docking/priming steps are reversible such that SV states maintain a dynamic equilibrium,

3. only SVs equipped with a mature release machinery, i.e. in the tightly docked (TS) or labile tightly docked (TSL) state, are fusion-competent,

4. the rate constants for the ES→LS transition (k_1) and for the LS→TS transition (k_2) are Ca^{2+}-dependent while all other rate constants have fixed values,

5. immediately following a SV fusion event, release sites are in a refractory state (ERS) and become available for refilling (ES) with a docked SV with first-order kinetics (b_4), and

6. the refilling of vacant release sites is assumed to utilize an infinite replenishment pool.

These model properties imply that a release site at any given time t can be either empty and available for docking/priming (N_{ES}), occupied by a docked/primed SV, or empty and unavailable for docking/priming (N_{ERS}). SV docking and priming proceed through two sequential maturation states: SVs first become loosely docked (LS SVs) before they mature into tightly docked fusion-competent vesicles (TS SVs). A small fraction (κ) of LS SVs transitions into a labile fusion-competent state (TSL SVs) immediately after each AP. TSL SVs quickly revert to the LS state due to a high backward rate constant for the LS←TSL transition (b_3). Thus, the entire pool of docked/primed SVs at a given time t can be subdivided into LS, TS and TSL SV subpools (SP_{LS}, SP_{TS}, SP_{TSL}, respectively), such that

$$N_{total} = N_{ES}(t) + SP_{LS}(t) + SP_{TS}(t) + SP_{TSL}(t) + N_{ERS}(t) \quad (1)$$

The following coupled ordinary differential equations describe temporal changes in state occupancies at rest and during ISIs:

$$\frac{d}{dt}N_{ES}(t) = -k_1 \cdot N_{ES}(t) + b_1 \cdot SP_{LS}(t) + b_4 \cdot N_{ERS} \quad (2)$$

$$\frac{d}{dt}SP_{LS}(t) = -(b_1 + k_2) \cdot SP_{LS}(t) + b_2 \cdot SP_{TS}(t) + b_3 \cdot SP_{TSL}(t) + k_1 \cdot N_{ES}(t) \quad (3)$$

$$\frac{d}{dt} SP_{TS}(t) = -b_2 \cdot SP_{TS}(t) + k_2 \cdot SP_{LS}(t) \quad (4)$$

$$\frac{d}{dt} SP_{TSL}(t) = -b_3 \cdot SP_{TSL}(t) \quad (5)$$

$$\frac{d}{dt} N_{ERS}(t) = -b_4 \cdot N_{ERS}(t) \quad (6)$$

Note that $SP_{TSL} = 0$ for resting synapses and after $ISIs \gg 1/b_3$.

This system of coupled ordinary differential equations (eqn. 2–6) was solved numerically using Igor Pro's built-in routine 'IntegrateODE' and choosing a fifth-order Runge-Kutta-Fehlberg algorithm. The backward rate constants b_1, b_2, b_3 and b_4 have fixed values, and the two forward rate constants k_1 and k_2 are modeled as Ca^{2+}-dependent quantities which increase linearly with the 'effective' $[Ca^{2+}]_i$ ($[Ca^{2+}](t)'$) according to:

$$k_1(t) = k_{1,rest} + \sigma_1 \cdot ([Ca^{2+}](t)' - [Ca^{2+}]_{rest}) \quad (7)$$

$$k_2(t) = k_{2,rest} + \sigma_2 \cdot ([Ca^{2+}](t)' - [Ca^{2+}]_{rest}). \quad (8)$$

$[Ca^{2+}]_{rest}$ represents the resting $[Ca^{2+}]_i$, which was assumed to be 50 nM under control conditions. σ_1 and σ_2 are linear slope factors characterizing the Ca^{2+} dependence of the SV priming steps.

For each SV fusion event, the quantal content m_j of the $EPSC_j$ triggered by stimulus j was calculated as the product of $p_{fusion,j} \cdot (SP_{TS}(t_j) + SP_{TSL}(t_j))$ with subpool sizes and p_{fusion} evaluated immediately before stimulus arrival. Index j indicates stimulus index, j = 1–40. SP_{TS} and SP_{TSL} were decremented by their contribution to m_j and N_{ERS} was incremented by m_j.

The value of $p_{fusion,j}$, the fusion probability at arrival of the jth AP, was determined according to:

$$p_{fusion,j} = p_{fusion,1} \cdot {y_j}^{4.5} \cdot z_j \quad (9)$$

with $y \geq 1$ and $z \leq 1$. Here, $p_{fusion,1}$ designates the fusion probability for the first eEPSC in a train (table S1), y_j accounts for changes in local $[Ca^{2+}]$ during repetitive stimulation ($y_j =$

$[Ca^{2+}]_j/[Ca^{2+}]_1$). The parameter z accounts for a reduction of p_{fusion} during repetitive stimulation, as indicated by NTF analysis, which, however, was relatively small.

Both variables y_j and z_j were initialized to 1 at the onset of a stimulus train. The variable y was incremented after each AP by

$$y_{inc} = y_{inc,1} \cdot (y_{max} - y_j) \tag{10}$$

and z was decremented by

$$z_{dec} = z_{dec,1} \cdot (z_j - z_{min}) \tag{11}$$

During ISIs, both $y(t)$ and $z(t)$ were solved numerically, together with other model parameters, according to

$$\frac{d}{dt}\, y(t) = (1 - y(t)) \cdot k_y \tag{12}$$

$$\frac{d}{dt}\, z(t) = (1 - z(t)) \cdot k_z \tag{13}$$

The presynaptic 'effective' Ca^{2+} transient $[Ca^{2+}](t)'$, governing the Ca^{2+}-dependent acceleration of SV priming, was assumed to decay back to its resting value $[Ca^{2+}]_{rest}$ with a triple-exponential time course, reflecting a Ca^{2+} transient composed of a fast-decaying local transient ($\Delta[Ca^{2+}]_l'$) plus a slowly-decaying global transient ($\Delta[Ca^{2+}]_g'$):

$$\begin{aligned}[Ca^{2+}](t)' = {} & \Delta[Ca^{2+}]_l' \cdot \exp\left(\frac{-(t - t_j)}{\tau_{fast}}\right) + \\ & \Delta[Ca^{2+}]_g' \cdot f_{fast} \cdot \exp\left(\frac{-(t - t_j)}{\tau_{fast}}\right) + \\ & \Delta[Ca^{2+}]_g' \cdot (1 - f_{fast}) \cdot \exp\left(\frac{-(t - t_j)}{\tau_{slow}}\right) + [Ca^{2+}]_{rest}\end{aligned} \tag{14}$$

The shape of $[Ca^{2+}](t)'$ may be interpreted as the time course of the Ca^{2+}-bound fraction of Ca^{2+} sensors, possibly weighted by their relative contribution to activity-dependent SV priming (*100*). Manually optimized model parameter values for wt/− and HK/− synapses are compared in table S2.

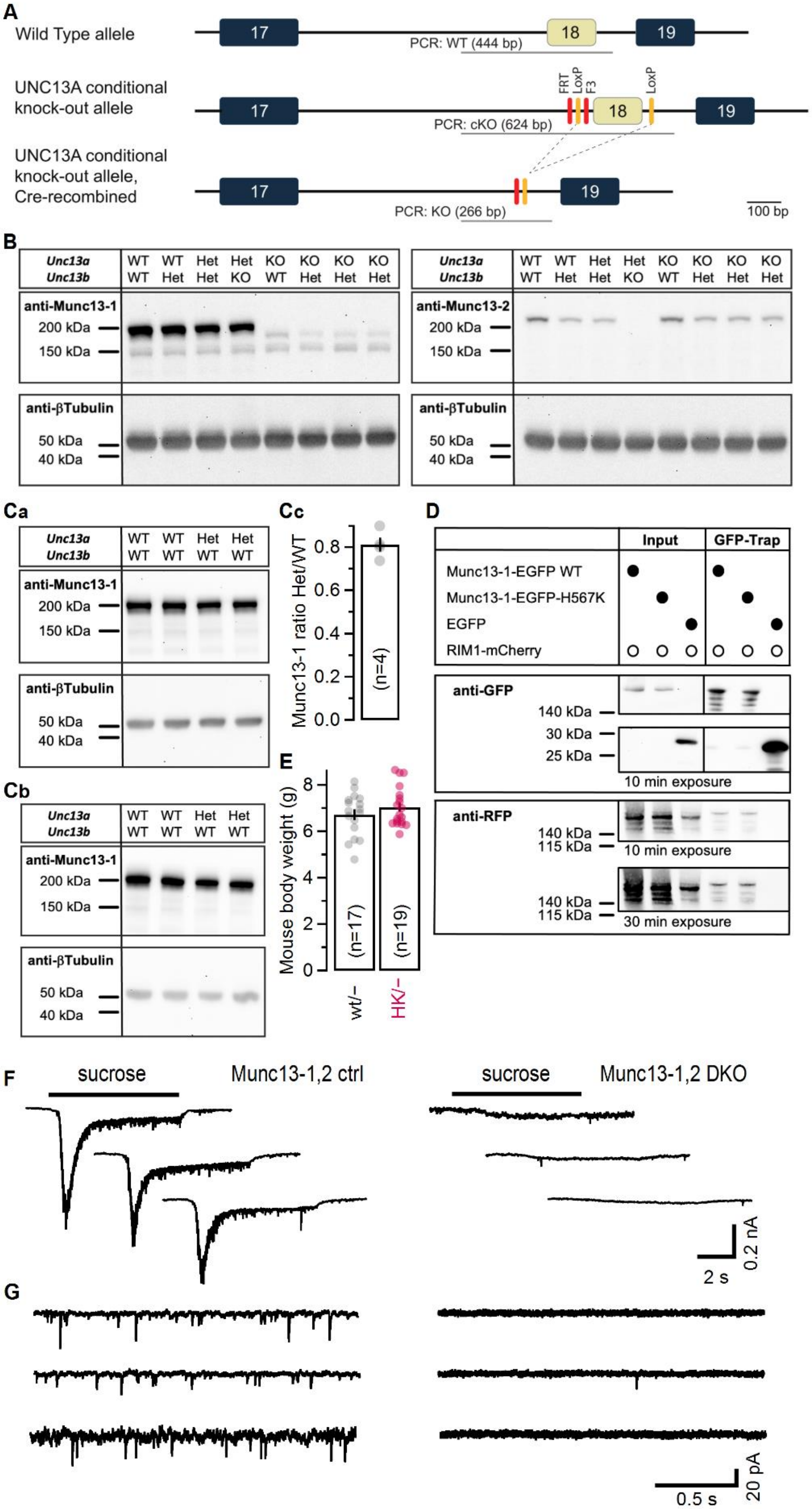
A
Wild Type allele
17
18
19
PCR: WT (444 bp)
UNC13A conditional knock-out allele
FRT
LoxP
F3
LoxP
PCR: cKO (624 bp)
UNC13A conditional knock-out allele, Cre-recombined
PCR: KO (266 bp)
100 bp
B
Unc13a
Unc13b
WT WT Het Het KO KO KO KO
WT Het Het KO WT Het Het Het
anti-Munc13-1
200 kDa
150 kDa
anti-βTubulin
50 kDa
40 kDa
anti-Munc13-2
Ca
Cb
Cc
Munc13-1 ratio Het/WT
(n=4)
D
Input
GFP-Trap
Munc13-1-EGFP WT
Munc13-1-EGFP-H567K
EGFP
RIM1-mCherry
anti-GFP
140 kDa
30 kDa
25 kDa
10 min exposure
anti-RFP
115 kDa
30 min exposure
E
Mouse body weight (g)
(n=17)
(n=19)
wt/−
HK/−
F
sucrose
Munc13-1,2 ctrl
Munc13-1,2 DKO
0.2 nA
2 s
G
20 pA
0.5 s

Figure S1. Generation and validation of conditional Munc13-1 knockout mice.

(A) Schematic representation of the Unc13a conditional knockout strategy in this study. In the cKO allele, LoxP sites flank exon 18. To confirm that the deletion of exon 18 leads to a complete loss of Munc13-1 expression, an EIIA-Cre driver line was used to mediate recombination, leading to excision of exon 18 and the generation of the KO allele. This excision was verified through PCR analysis using specific primers that produce distinct fragment sizes: WT (444 bp), cKO (624 bp), and KO (266 bp). Using this strategy, heterozygous Munc13-1 constitutive KO mice were generated and subsequently crossbred with homozygous Munc13-2 constitutive KO mice. The resulting offspring were heterozygous for both Munc13-1 and Munc13-2, and interbreeding of these mice produced Munc13-1,2 DKO mice.

(B) Absence of Munc13-1 and Munc13-2 expression following gene deletion as described in (**A**), verified by Western blotting of brains from eight E18 mouse embryos. Mouse genotypes are indicated in the top row. Munc13-1 expression was undetectable in Munc13-$1^{-/-}$ brains and slightly reduced in Munc13-$1^{wt/-}$ brains. Amount of protein loaded was 20 µg per lane.

(C) Western blot analysis of Munc13-1 protein expression in cortices obtained from Munc13-$1^{wt/wt}$ and Munc13-$1^{wt/-}$ postnatal day (P) 19–21 mouse pups. Mouse genotypes are indicated in the top rows. Amount of protein loaded was 10 µg per lane. Four littermate pups were evaluated in each of the two Western blots at P21 (**Ca**) and P19 (**Cb**). Munc13-1 expression levels were normalized to those of β-Tubulin, which served as loading control. Munc13-1 expression was reduced to 81.1 ± 3.3% (n=4) in Munc13-$1^{wt/-}$ cortices as compared to littermate Munc13-$1^{wt/wt}$ (**Cc**).

(D) Co-immunoprecipitation experiment assessing the interaction of RIM1 with Munc13-1^{wt} and Munc13-1^{HK}. The wt and HK mutant of Munc13-1-EGFP were expressed with RIM1-mCherry in HEK293FT cells. GFP-trap agarose beads were used to capture Munc13-1, and co-immunoprecipitation of RIM1-mCherry was evaluated using a Western blot analysis with an anti-RFP antibody (to detect RIM1-mCherry) or an anti-GFP antibody (to confirm the pulldown of the EGFP variants). EGFP expressed together with RIM1-mCherry was used as a negative control. 0.05% of the input samples and 10% of the eluate samples were loaded per lane.

(E) Scatter dot plots and bar graphs showing individual and mean values of body weights of P15–P18 brainstem-specific heterozygous Munc13-1 wt/− and HK/− mice. Numbers of pups are given in parenthesis.

Error bars in (Cc) and (E) indicate SEM.

(F) Sample traces of current responses induced by a hypertonic sucrose solution (500 mM, 7 s) in glutamatergic neurons from ctrl and Munc13-1,2 DKO hippocampal cultures, illustrating the absence of the readily releasable vesicle pool in DKO synapses. This functional characterization is consistent with data obtained using the conventional Munc13-1 KO mouse when crossed with Munc13-2 KO mice (*112*), indicating that deletion of Munc13 expression via the deletion of exon 18 eliminates Munc13-1 protein expression. The control group (ctrl) consisted of pups with the following genotypes: Munc13-1$^{wt/wt}$ Munc13-2$^{wt/-}$, Munc13-1$^{wt/-}$ Munc13-2$^{wt/-}$ and Munc13-1$^{wt/-}$ Munc13-2$^{wt/wt}$.

(G) Sample traces of spontaneously occurring miniature excitatory postsynaptic currents (mEPSCs) recorded in ctrl and Munc13-1,2 DKO hippocampal neurons.

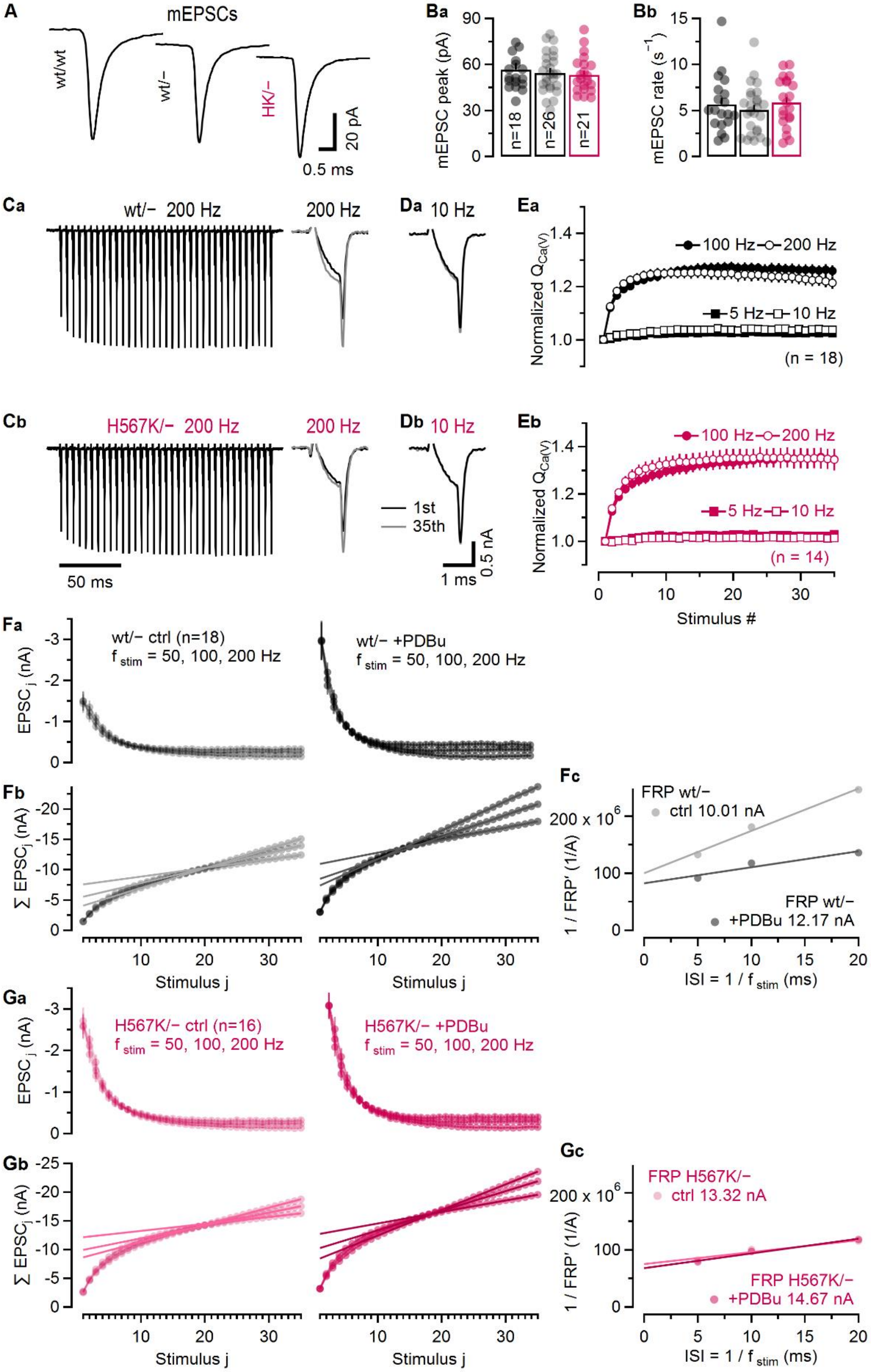

A
mEPSCs
wt/wt
wt/−
HK/−
20 pA
0.5 ms
Ba
mEPSC peak (pA)
n=18
n=26
n=21
Bb
mEPSC rate (s⁻¹)
Ca
wt/− 200 Hz
200 Hz
Da
10 Hz
Ea
100 Hz
200 Hz
5 Hz
10 Hz
Normalized Q Ca(V)
(n = 18)
Cb
H567K/− 200 Hz
200 Hz
Db
10 Hz
1st
35th
0.5 nA
1 ms
50 ms
Eb
100 Hz
200 Hz
5 Hz
10 Hz
(n = 14)
Stimulus #
Fa
wt/− ctrl (n=18)
f stim = 50, 100, 200 Hz
wt/− +PDBu
f stim = 50, 100, 200 Hz
EPSC j (nA)
Fb
∑ EPSC j (nA)
Stimulus j
Fc
FRP wt/−
ctrl 10.01 nA
FRP wt/−
+PDBu 12.17 nA
200 x 10⁶
1 / FRP' (1/A)
ISI = 1 / f stim (ms)
Ga
H567K/− ctrl (n=16)
f stim = 50, 100, 200 Hz
H567K/− +PDBu
f stim = 50, 100, 200 Hz
Gb
Gc
FRP H567K/−
ctrl 13.32 nA
FRP H567K/−
+PDBu 14.67 nA

Figure S2. mEPSC properties, changes in presynaptic Ca^{2+} influx during trains of AP-like step depolarizations and PDBu-induced changes in FRP size in wt/− and HK/− calyx terminals.

(A) Representative average mEPSC waveforms recorded in the absence of kyn in a wt/wt (*left*), a wt/− (*middle*) and an HK/− (*right*) synapse. Average mEPSC waveforms were obtained from >800 individual mEPSCs in each synapse, recorded in the presence of 5 µM strychnine.

(B) Bar graphs and scatter dot plots showing average values and individual data points, respectively, of mEPSC amplitudes (**Ba**) and mEPSC rates (**Bb**) in wt/wt (*left*, *dark gray*), wt/− (*middle, light gray*) and HK/− (*right, magenta*) synapses.

(C) Modulation of calyceal $I_{Ca(V)}$ (*left*) during 200 Hz trains of brief AP-like depolarization (thirty-five 1-ms steps from V_h = −80 to 0 mV) recorded in a wt/− (**Ca**) and an HK/− (**Cb**) calyx. The last (*gray*) and the first (*black*) $I_{Ca(V)}$ are shown superimposed for comparison at an expanded timescale (*right*). During short high-frequency (100 or 200 Hz) trains of AP-like presynaptic depolarizations, calyceal $I_{Ca(V)}$ facilitates, whereas nearly constant $I_{Ca(V)}$ amplitudes are measured during low-frequency trains (5 or 10 Hz) in post-hearing wt/− and HK/− calyces.

(D) Same experiments as in (**C**) but using 10 Hz trains. The last (*gray*) and the first (*black*) $I_{Ca(V)}$ are superimposed.

(E) Summary plots showing mean normalized charge of $I_{Ca(V)}$ trains elicited by 5, 10, 100 and 200 Hz stimulus trains and recorded in wt/− (**Ea**) and HK/− (**Eb**) calyces.

(F) Mean amplitudes (**Fa**) and mean cumulative amplitudes (**Fb**) of eEPSC trains elicited by 50, 100 and 200 Hz (35 APs) stimulation and recorded in wt/− synapses before (*left*) and after (*right*) application of 1 µM PDBu in the presence of 1 mM kyn. 1/*FRP′* is plotted versus ISI for recordings in the absence and presence of PDBu (**Fc**). Solid lines represent linear regressions fitted to the scatterplots; regression intercepts estimate 1/*FRP*, corrected for incomplete pool depletion.

(G) Same experiments as in (**F**) but for HK/− synapses.

Numbers of terminals or synapses are given in parenthesis in (B), (E), (Fa) and (Ga).

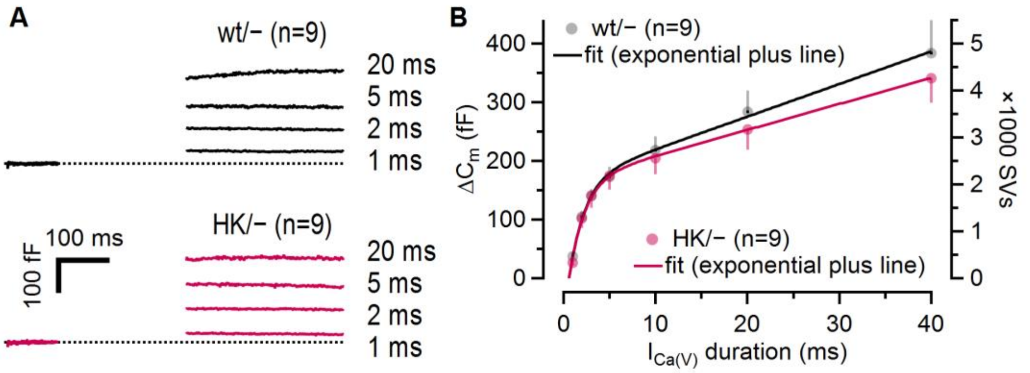

Figure S3. Synaptic vesicle pool depletion in wt/− and HK/− calyx terminals assayed by presynaptic capacitance measurements.

(A) Average presynaptic ΔC_m responses elicited by depolarizing voltage steps (from V_h = –80 to 0 mV) of four durations (1, 2, 5, and 20 ms, as indicated next to the traces) recorded in wt/− (*top*) and HK/− (*bottom*) calyces using a patch-pipette solution containing 0.1 mM EGTA.

(B) Summary plot of presynaptic ΔC_m (mean values measured between ~500 and ~550 ms after the onset of the depolarization) versus depolarization duration for wt/− and HK/− calyces. Solid lines represent least-squares fits using a function corresponding to the sum of an exponential (amplitudes ~168 fF and ~167 fF in wt/− and HK/−, respectively, corresponding to ~2097 SVs and ~2090 SVs assuming a single SV capacitance of 80 aF) plus a linear component (slopes ~5.7 fF/ms and ~ 4.4 fF/ms in wt/− and HK/−, respectively). The *FRP* is nearly completely depleted after a 5 ms-long depolarization. Vesicle fusion at longer step durations reflects release from the slowly-releasable pool (*SRP*) (*52*) and newly-recruited SVs, which cannot be reliably distinguished under these experimental conditions (*23*, *113*).

Error bars in (B) indicate SEM. Numbers of calyx terminals are indicated in parenthesis in (A, B).

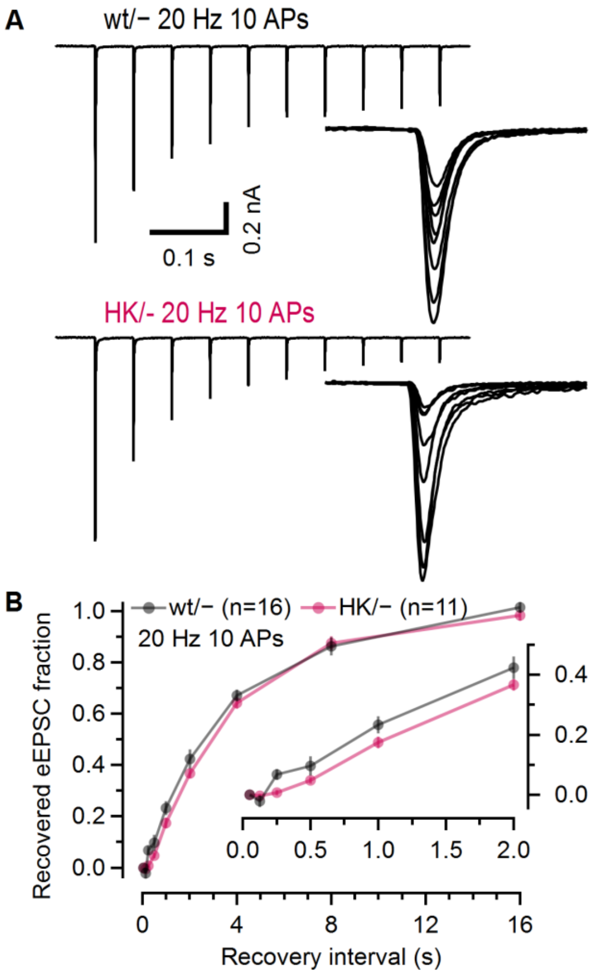

Figure S4. Recovery time course after 20 Hz stimulation in wt/− and HK/− calyx synapses. **(A)** Recovery of eEPSCs after 20 Hz (10 APs) stimulation was evaluated at intervals of 0.125, 0.25, 0.5, 1, 2, 4, 8 and 16 s. Sample traces of the 20 Hz eEPSC train (*left*) and test eEPSCs (*right*) at varying recovery intervals are shown for a wt/− (*top*) and an HK/− (*bottom*) synapse. **(B)** Mean time courses of eEPSC recovery are plotted for wt/− and HK/− synapses, with the first 2 s of recovery shown at an expanded timescale in the inset. The number of synapses tested is given in parenthesis.

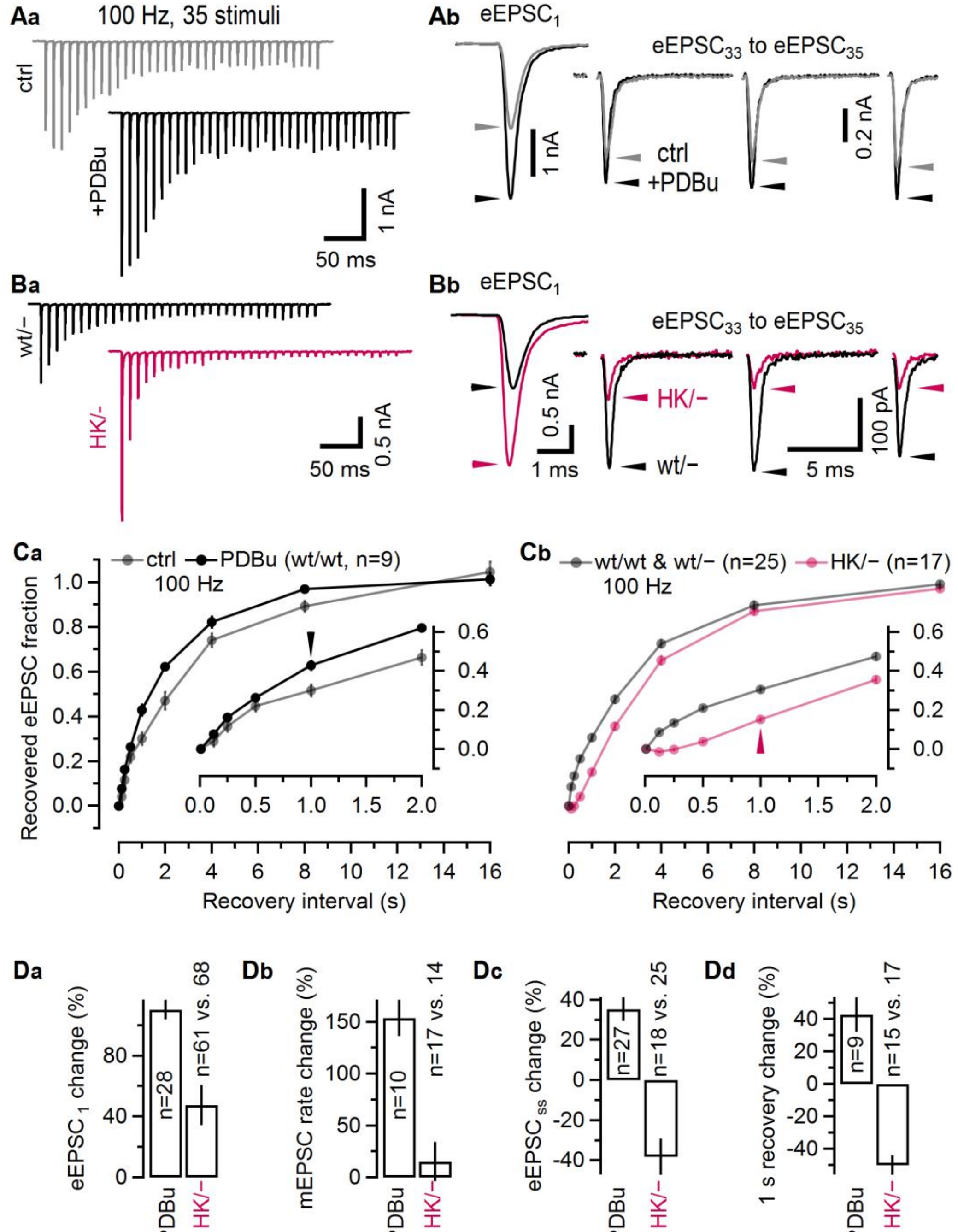
Aa
100 Hz, 35 stimuli
ctrl
+PDBu
1 nA
50 ms
Ab
eEPSC1
eEPSC33 to eEPSC35
1 nA
ctrl
+PDBu
0.2 nA
Ba
wt/−
HK/-
0.5 nA
50 ms
Bb
eEPSC1
eEPSC33 to eEPSC35
0.5 nA
1 ms
HK/−
wt/−
100 pA
5 ms
Ca
ctrl
PDBu (wt/wt, n=9)
100 Hz
Recovered eEPSC fraction
Recovery interval (s)
Cb
wt/wt & wt/− (n=25)
HK/− (n=17)
100 Hz
Recovery interval (s)
Da
eEPSC1 change (%)
n=28
n=61 vs. 68
PDBu
HK/−
Db
mEPSC rate change (%)
n=10
n=17 vs. 14
PDBu
HK/−
Dc
eEPSCss change (%)
n=27
n=18 vs. 25
PDBu
HK/−
Dd
1 s recovery change (%)
n=9
n=15 vs. 17
PDBu
HK/−

Figure S5. The HK C_1 mutation does not simply mimic the DAG/PDBu-bound state of the Munc13-1 C_1 domain because, unlike PDBu application, it decreases steady-state release during high-frequency trains and slows the eEPSC recovery kinetics.

(A) Sample traces of 100 Hz eEPSC trains (35 stimuli) recorded before (*gray*) and after (*black*) application of 1 µM PDBu in a wt/wt synapse (**Aa**). The initial eEPSCs (*left*) and the final three eEPSCs of the trains (*right*) are superimposed for comparison (**Ab**).

(B) Sample traces of 100 Hz eEPSC trains (35 stimuli) obtained under control conditions from a wt/wt (*black*) and an HK/− (*magenta*) synapse (**Ba**). The initial eEPSCs (*left*) and the final three eEPSCs of the trains (*right*) are superimposed for comparison (**Bb**). Despite affecting $eEPSC_1$ similarly, PDBu application enhances steady-state release, whereas the H567K C_1 mutation decreases it.

(C) Mean time courses of eEPSC recovery from depression induced by 100 Hz stimulation measured in either wt/wt synapses before (*gray*) and after (*black*) PDBu application (**Ca**), or in wt (pooled data from wt/wt and wt/−) and HK/− synapses (**Cb**). The first 2 s of the recovery timecourse are shown at an expanded timescale in the insets. PDBu application speeds up, whereas the H567K C_1 mutations slows down recovery.

(D) Comparison of relative changes induced by PDBu application (*left bars*) or by the HK C_1 mutation (*right bars*) for $eEPSC_1$ amplitudes (**Da**), mEPSC rates (**Db**), steady-state eEPSCs during 100 Hz trains (**Dc**), and recovery from depression measured at 1 s recovery interval (**Dd**). While increasing $eEPSC_1$ amplitudes, the H567K C_1 mutation has little effect on mEPSC rates, but decreases $eEPSC_{ss}$ and slows recovery of eEPSCs from depression after high-frequency stimulation.

Percentage changes in (**D**) were calculated according to $(p_2/p_1 - 1) \times 100$, where p_1 is the mean parameter value in wt synapses under control conditions, and p_2 is the respective mean value either after PDBu application (*left bars*) or in HK/− synapses (*right bars*). SEM estimates of the ratios of mean values in (**D**) were derived by Gaussian error propagation. $p<0.0001$ for (Da) to (Dd) (two-sample *z*-test using error-propagated SEMs).

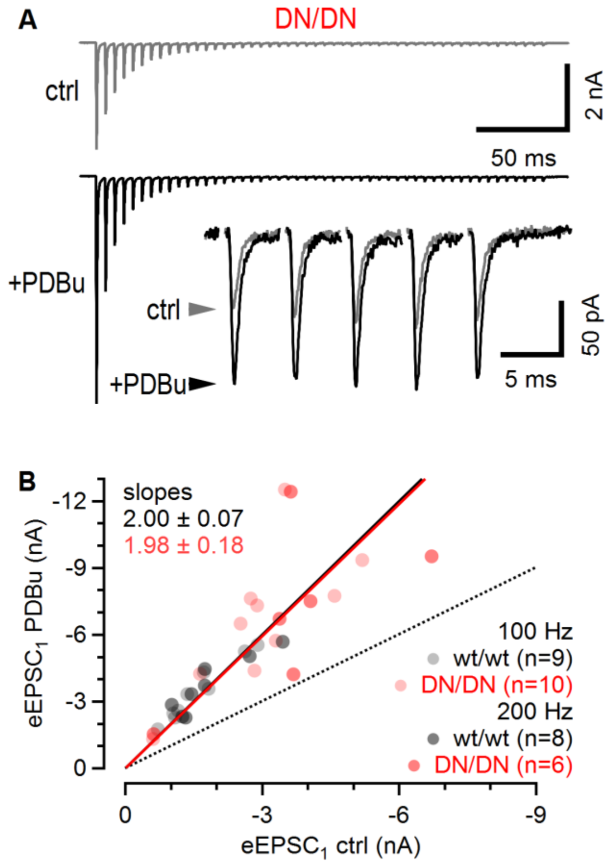

Figure S6. PDBu-induced potentiation of initial and steady-state eEPSCs is unperturbed in DN/DN calyx synapses.

(A) Sample traces of 200 Hz eEPSC trains (50 stimuli) obtained under control conditions (*top*, *gray*) and in the presence of 1 µM PDBu (*bottom*, *black*) in a DN/DN synapse. The inset shows the last 5 eEPSCs at an expanded timescale.

(B) Scatterplot of $eEPSC_1$ recorded in the presence of 1 µM PDBu versus the respective eEPSCs recorded under control conditions for wt/wt and DN/DN synapses. The dotted and solid lines represent the identity line and linear regressions through the origin, respectively. Regression slopes measure eEPSC potentiation in the presence of PDBu and were nearly identical for wt/wt and DN/DN synapses.

Numbers of synapses are given in parenthesis in (B).

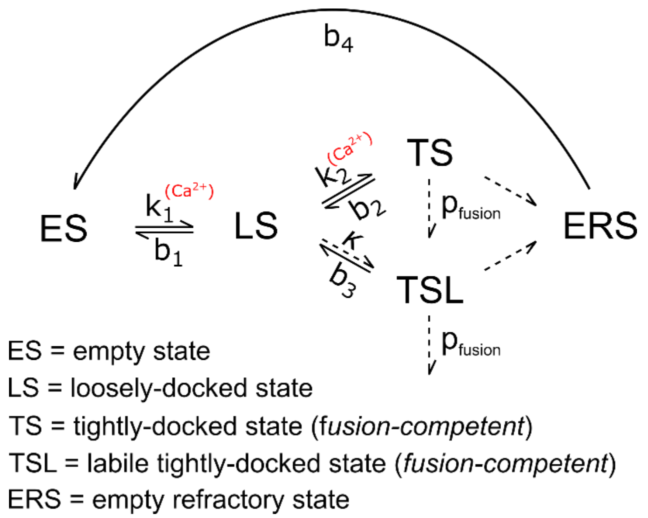

Figure S7. Kinetic scheme for the numerical simulation of short-term plasticity. Sequential scheme for SV priming and fusion. SVs dock to an empty release site (ES) and undergo two priming steps to sequentially transition to the states LS (loosely-docked) and TS (tightly-docked). An additional labile tightly-docked state (TSL) is required for reproducing experimental data obtained in response to high-frequency stimulation (f_{stim} ≥50 Hz). Only SVs in state TS and TSL are fusion-competent. Although SVs residing in TS or TSL have the same p_{fusion}, the two states differ with respect to their stability. While TS has a lifetime in the range of 3–4 s, TSL relaxes back to LS within about $1/b_3 = 70$ ms. Release sites vacated after a SV fusion event reside for some time in a refractory empty state (ERS). State transitions represented by dashed lines indicate instantaneous transitions, while those represented by solid lines occur with rate constants as shown. The rate constants k_1 and k_2 are regulated by $[Ca^{2+}]_i$ (see also table S2).

Table S1: Functional parameters of synaptic transmission (related to Figs. 1, 2, 4, 5)

Parameter	wt/−	HK/−	statistical significance#
**single eEPSCs** (in 1 mM kyn)	n=61	n=68	
amplitude (nA)	−1.69 ± 0.12	−2.49 ± 0.14	$p < 0.001$
half-width (ms)	0.48 ± 0.009	0.48 ± 0.008	n.s.
20-80% rise time (ms)	0.14 ± 0.002	0.14 ± 0.001	n.s.
FRP (in 2 mM γ-DGG)	n=18	n=25	
size (SVs)§	2519 ± 437	2506 ± 172	n.s.
F§	0.10 ± 0.01	0.18 ± 0.01	$p < 0.001$
f_{TS}&	0.28 ± 0.02	0.46 ± 0.02	$p < 0.001$
**mEPSCs** (w/o kyn)	n=26	n=21	
frequency (s^{-1})	5.05 ± 0.52	5.89 ± 0.58	n.s.
amplitude (pA)	54.6 ± 2.6	53.3 ± 2.1	n.s.
half-width (ms)	0.33 ± 0.007	0.31 ± 0.01	n.s.
20-80% rise time (ms)	0.09 ± 0.001	0.09 ± 0.002	n.s.
**presynaptic $I_{Ca(V)}$ and ΔC_m**	n=18	n=19	
peak amplitude during 10 ms step from V_h −80 to 0 mV (nA)	−1.332 ± 0.147	−1.255 ± 0.098	n.s.
terminal capacitance (pF)	14.3 ± 1.22	13.89 ± 0.85	n.s.
	n=15	n=19	
ΔC_m in response to 10 ms step from V_h −80 to 0 mV (fF)	205.6 ± 25.62	183.14 ± 18.99	n.s.
**fractional eEPSC recovery from depression** (in 1 mM kyn; 100 Hz)	n=15	n=17	
interval = 0.125 s	0.09 ± 0.014	−0.0135 ± 0.005	$p < 0.001$
interval = 0.25 s	0.116 ± 0.016	−0.001 ± 0.009	$p < 0.001$
interval = 0.5 s	0.208 ± 0.018	0.039 ± 0.011	$p < 0.001$
interval = 1 s	0.301 ± 0.015	0.151 ± 0.015	$p < 0.001$
interval = 2 s	0.462 ± 0.025	0.355 ± 0.019	$p = 0.0025$
interval = 4 s	0.718 ± 0.028	0.65 ± 0.024	n.s.
interval = 8 s	0.868 ± 0.029	0.87 ± 0.02	n.s.
interval = 16 s	0.982 ± 0.019	0.97 ± 0.014	n.s.
**fractional eEPSC recovery from depression** (in 1 mM kyn; 200 Hz)	n=15	n=19	
interval = 0.125 s	0.067 ± 0.013	−0.024 ± 0.007	$p < 0.001$
interval = 0.25 s	0.141 ± 0.018	−0.016 ± 0.008	$p < 0.001$
interval = 0.5 s	0.213 ± 0.016	0.021 ± 0.01	$p < 0.001$
interval = 1 s	0.314 ± 0.018	0.125 ± 0.013	$p < 0.001$
interval = 2 s	0.458 ± 0.018	0.323 ± 0.016	$p < 0.001$
interval = 4 s	0.687 ± 0.021	0.642 ± 0.016	n.s.
interval = 8 s	0.916 ± 0.02	0.88 ± 0.012	n.s.

interval = 16 s	0.995 ± 0.022	0.988 ± 0.013	n.s.
**fractional eEPSC recovery from depression** (in 1 mM kyn; 10 Hz)	n=16	n=11	
interval = 0.125 s	0.021 ± 0.017	0.004 ± 0.009	n.s.
interval = 0.25 s	0.051 ± 0.015	0.023 ± 0.01	n.s.
interval = 0.5 s	0.109 ± 0.014	0.064 ± 0.017	n.s.
interval = 1 s	0.244 ± 0.032	0.171 ± 0.018	n.s.
interval = 2 s	0.445 ± 0.029	0.407 ± 0.024	n.s.
interval = 4 s	0.674 ± 0.031	0.672 ± 0.028	n.s.
interval = 8 s	0.903 ± 0.02	0.871 ± 0.025	n.s.
interval = 16 s	0.995 ± 0.02	1.003 ± 0.031	n.s.
**fractional eEPSC recovery from depression** (in 1 mM kyn; 20 Hz)	n=16	n=11	
interval = 0.125 s	-0.022 ± 0.020	-0.004 ± 0.006	n.s.
interval = 0.25 s	0.066 ± 0.017	0.007 ± 0.009	n.s.
interval = 0.5 s	0.096 ± 0.031	0.047 ± 0.011	n.s.
interval = 1 s	0.232 ± 0.027	0.173 ± 0.02	n.s.
interval = 2 s	0.422 ± 0.037	0.366 ± 0.02	n.s.
interval = 4 s	0.671 ± 0.023	0.641 ± 0.023	n.s.
interval = 8 s	0.862 ± 0.035	0.874 ± 0.028	n.s.
interval = 16 s	1.014 ± 0.021	0.983 ± 0.021	n.s.
**eEPSC amplitude potentiation by PDBu** (in 1 mM kyn; fold change)	n=19	n=17	
$eEPSC_1$	2.21 ± 0.11	1.29 ± 0.05	$p < 0.001$
$eEPSC_{ss}$ (100 & 200 Hz stimulation)	1.49 ± 0.06	1.58 ± 0.07	n.s.
**mEPSC rate potentiation by PDBu** (w/o kyn; fold change)	n=10	n=10	
mEPSC rate	2.61 ± 0.19	1.28 ± 0.09	$p < 0.001$
**eEPSC amplitude potentiation by 7,8-DHF** (in 1 mM kyn; fold change)	n=11	n=10	
$eEPSC_1$	1.35 ± 0.034	1.14 ± 0.035	$p = 0.004$

[#] If not stated otherwise, a two-tailed Welch–Satterthwaite *t*-test was used to test for statistical significance of the differences between sample means.
[§] Estimated by analysis of cumulative release during high-frequency stimulation (50, 100, 200 and 333 Hz, 40 stimuli; Fig. 4, E and F). Error estimates obtained through balanced bootstrapping.
[&] NTF-based estimate (Fig. 4G).
n.s. = not statistically significant

Table S2: Parameters for modelling STP and eEPSC recovery during and after stimulus train, respectively, in wt/− and HK/− calyx synapses (related to Fig. 6)

**Model parameter**	**wt/−**	**HK/−**	**Change#**
Global $\Delta[Ca^{2+}]_i$ τ_{fast} (s)	0.05	0.05	
Global $\Delta[Ca^{2+}]_i$ τ_{slow} (s)	0.24	0.24	
Global $\Delta[Ca^{2+}]_i$ amplitude fraction_{slow}	0.12	0.12	
Global $\Delta[Ca^{2+}]_i$ amplitude (M)	3.9e−07	3.9e−07	
Local $\Delta[Ca^{2+}]_i$ τ (s)	0.00036	0.00036	
Local $\Delta[Ca^{2+}]_i$ amplitude (M)	3.3e−05	3.3e−05	
Resting $[Ca^{2+}]_i$ (M)	5.00e−08	5.00e−08	
p_{fusion}	0.341	0.379	+11%
N_{total} (SVs)	3300	3150	−4.5%
$k_{1,rest}$ (s^{-1})	0.500	0.419	−16%
b_1 (s^{-1})	0.231	0.194	−16%
**σ_1 ($M^{-1}\cdot s^{-1}$)**	**859000**	**221000**	**−74%**
**$k_{2,rest}$ (s^{-1})**	**0.1357**	**0.2916**	**+115%**
b_2 (s^{-1})	0.3514	0.3514	
**σ_2 ($M^{-1}\cdot s^{-1}$)**	**1.22e+06**	**1.81e+06**	**+48%**
$\tau_{TSL} = 1/b_3$ (s)	0.07	0.07	
κ (TSL fraction)	0.08	0.08	
b_4 (s^{-1})	3.3	3.3	
y_{inc}	0.36	0.36	
z_{dec}	0.4	0.4	
y_{max}	1.22	1.22	
z_{min}	0.77	0.77	
τ_y (s)	0.014	0.014	
τ_z (s)	3	3	

Increases and decreases in model parameter values are indicated by blue and red color, respectively. Model parameter that differ by >20% between wt/− and HK/− synapses are indicated in bold font.

Table S3: Post-tetanic potentiation of eEPSC amplitudes and mEPSC rates (related to Figs. 7, 8)

Parameter	wt/−	HK/−	HK/DN	statistical significance
**eEPSC amplitude potentiation** (in 1 mM kyn; fold change)	n=26	n=29	n=8	
$eEPSC_{PTP}$ / $eEPSC_{ctrl}$	2.08 ± 0.08	1.24 ± 0.04	1.37 ± 0.07	p < 0.0001 (wt/− vs HK/−)[&] p < 0.0001 (wt/− vs HK/DN)[&] p = 0.52761 (HK/− vs HK/DN)[&]
remaining PTP (relative to wt/−)		(1.24−1)/(2.08−1)×100% = 22%	(1.37−1)/(2.08−1)×100% = 34%	
**mEPSC rate potentiation** (w/o kyn; fold change)	n=10	n=7	n.a.	
$rate_{PTP}$ / $rate_{ctrl}$	6.98 ± 1.26	4.73 ± 0.63	n.a.	n.s.[#]
	**wt/wt**[§]	**DN/DN**	**KW/KW**	statistical significance[#]
**eEPSC amplitude potentiation** (in 1 mM kyn; fold change)	n=34	n=23	n=16	
$eEPSC_{PTP}$ / $eEPSC_{ctrl}$	2.07 ± 0.09	1.54 ± 0.05	2.92 ± 0.24	p = 0.00438 (wt/wt vs DN/DN)[&] p < 0.001 (wt/wt vs KW/KW)[&]
remaining PTP (relative to wt/wt)		(1.54−1)/(2.07−1)×100% = 50%		
**mEPSC rate potentiation** (w/o kyn; fold change)	n=9	n.a.	n=10	
$rate_{PTP}$ / $rate_{ctrl}$	6.46 ± 0.86	n.a.	6.57 ± 0.65	n.s.[#]

[§] Pooled results obtained from wt/wt littermates of the DN or the KW mouse line.
[#] Two-tailed Welch–Satterthwaite *t*-test
[&] Tukey's multiple comparisons of means
n.a. = not available; n.s. = not statistically significant

REFERENCES

1. T. C. Südhof, A molecular machine for neurotransmitter release: Synaptotagmin and beyond. *Nat. Med.* **19**, 1227–1231 (2013).

2. J. Rizo, Molecular mechanisms underlying neurotransmitter release. *Annu. Rev. Biophys.* **51**, 377–408 (2022).

3. T. C. Südhof, The presynaptic active zone. *Neuron* **75**, 11–25 (2012).

4. M. Verhage, J. B. Sorensen, Vesicle docking in regulated exocytosis. *Traffic* **9**, 1414–1424 (2008).

5. I. Augustin, C. Rosenmund, T. C. Südhof, N. Brose, Munc13-1 is essential for fusion competence of glutamatergic synaptic vesicles. *Nature* **400**, 457–461 (1999).

6. L. Siksou, F. Varoqueaux, O. Pascual, A. Triller, N. Brose, S. Marty, A common molecular basis for membrane docking and functional priming of synaptic vesicles. *Eur. J. Neurosci.* **30**, 49–56 (2009).

7. B. Quade, M. Camacho, X. Zhao, M. Orlando, T. Trimbuch, J. Xu, W. Li, D. Nicastro, C. Rosenmund, J. Rizo, Membrane bridging by Munc13-1 is crucial for neurotransmitter release. *eLife* **8**, e42806 (2019).

8. M. Padmanarayana, H. Liu, F. Michelassi, L. Li, D. Betensky, M. J. Dominguez, R. B. Sutton, Z. Hu, J. S. Dittman, A unique C2 domain at the C terminus of Munc13 promotes synaptic vesicle priming. *Proc. Natl. Acad. Sci. U.S.A.* **118**, e2016276118 (2021).

9. J. E. Richmond, R. M. Weimer, E. M. Jorgensen, An open form of syntaxin bypasses the requirement for UNC-13 in vesicle priming. *Nature* **412**, 338–341 (2001).

10. Y. Lai, U. B. Choi, J. Leitz, H. J. Rhee, C. Lee, B. Altas, M. Zhao, R. A. Pfuetzner, A. L. Wang, N. Brose, J. Rhee, A. T. Brunger, Molecular mechanisms of synaptic vesicle priming by Munc13 and Munc18. *Neuron* **95**, 591–607.e10 (2017).

11. C. Ma, W. Li, Y. Xu, J. Rizo, Munc13 mediates the transition from the closed syntaxin-Munc18 complex to the SNARE complex. *Nat. Struct. Mol. Biol.* **18**, 542–549 (2011).

12. H. Sakamoto, T. Ariyoshi, N. Kimpara, K. Sugao, I. Taiko, K. Takikawa, D. Asanuma, S. Namiki, K. Hirose, Synaptic weight set by Munc13-1 supramolecular assemblies. *Nat. Neurosci.* **21**, 41–49 (2018).

13. M. R. Karlocai, J. Heredi, T. Benedek, N. Holderith, A. Lorincz, Z. Nusser, Variability in the Munc13-1 content of excitatory release sites. *eLife* **10**, e67468 (2021).

14. J. E. Johnson, J. Giorgione, A. C. Newton, The C1 and C2 domains of protein kinase C are independent membrane targeting modules, with specificity for phosphatidylserine conferred by the C1 domain. *Biochemistry* **39**, 11360–11369 (2000).

15. T. Hori, Y. Takai, T. Takahashi, Presynaptic mechanism for phorbol ester-induced synaptic potentiation. *J. Neurosci.* **19**, 7262–7267 (1999).

16. R. C. Malenka, D. V. Madison, R. A. Nicoll, Potentiation of synaptic transmission in the hippocampus by phorbol esters. *Nature* **321**, 175–177 (1986).

17. K. D. Parfitt, D. V. Madison, Phorbol esters enhance synaptic transmission by a presynaptic, calcium-dependent mechanism in rat hippocampus. *J. Physiol.* **471**, 245–268 (1993).

18. J. S. Rhee, A. Betz, S. Pyott, K. Reim, F. Varoqueaux, I. Augustin, D. Hesse, T. C. Südhof, M. Takahashi, C. Rosenmund, N. Brose, β phorbol ester- and diacylglycerol-induced augmentation of transmitter release is mediated by Munc13s and not by PKCs. *Cell* **108**, 121–133 (2002).

19. K. D. Wierda, R. F. Toonen, H. de Wit, A. B. Brussaard, M. Verhage, Interdependence of PKC-dependent and PKC-independent pathways for presynaptic plasticity. *Neuron* **54**, 275–290 (2007).

20. J. Waters, S. J. Smith, Phorbol esters potentiate evoked and spontaneous release by different presynaptic mechanisms. *J. Neurosci.* **20**, 7863–7870 (2000).

21. J. Basu, A. Betz, N. Brose, C. Rosenmund, Munc13-1 C1 domain activation lowers the energy barrier for synaptic vesicle fusion. *J. Neurosci.* **27**, 1200–1210 (2007).

22. C. F. Stevens, J. M. Sullivan, Regulation of the readily releasable vesicle pool by protein kinase C. *Neuron* **21**, 885–893 (1998).

23. K. H. Lin, M. Ranjan, N. Lipstein, N. Brose, E. Neher, H. Taschenberger, Number and relative abundance of synaptic vesicles in functionally distinct priming states determine synaptic strength and short-term plasticity. *J. Physiol.* **603**, 6135–6160 (2025).

24. S. Schotten, M. Meijer, A. M. Walter, V. Huson, L. Mamer, L. Kalogreades, M. ter Veer, M. Ruiter, N. Brose, C. Rosenmund, J. B. Sorensen, M. Verhage, L. N. Cornelisse, Additive effects on the energy barrier for synaptic vesicle fusion cause supralinear effects on the vesicle fusion rate. *eLife* **4**, e05531 (2015).

25. K. H. Lin, H. Taschenberger, E. Neher, A sequential two-step priming scheme reproduces diversity in synaptic strength and short-term plasticity. *Proc. Natl. Acad. Sci. U.S.A.* **119**, e2207987119 (2022).

26. M. Aldahabi, E. Neher, Z. Nusser, Different states of synaptic vesicle priming explain target cell type-dependent differences in neurotransmitter release. *Proc. Natl. Acad. Sci. U.S.A.* **121**, e2322550121 (2024).

27. N. Korogod, X. Lou, R. Schneggenburger, Presynaptic Ca^{2+} requirements and developmental regulation of posttetanic potentiation at the calyx of Held. *J. Neurosci.* **25**, 5127–5137 (2005).

28. D. Fioravante, Y. Chu, M. H. Myoga, M. Leitges, W. G. Regehr, Calcium-dependent isoforms of protein kinase C mediate posttetanic potentiation at the calyx of Held. *Neuron* **70**, 1005–1019 (2011).

29. D. Lee, K. H. Lee, W. K. Ho, S. H. Lee, Target cell-specific involvement of presynaptic mitochondria in post-tetanic potentiation at hippocampal mossy fiber synapses. *J. Neurosci.* **27**, 13603–13613 (2007).

30. R. L. Habets, J. G. Borst, Post-tetanic potentiation in the rat calyx of Held synapse. *J. Physiol.* **564**, 173–187 (2005).

31. H. Taschenberger, A. Woehler, E. Neher, Superpriming of synaptic vesicles as a common basis for intersynapse variability and modulation of synaptic strength. *Proc. Natl. Acad. Sci. U.S.A.* **113**, E4548–E4557 (2016).

32. D. Fioravante, W. G. Regehr, Short-term forms of presynaptic plasticity. *Curr. Opin. Neurobiol.* **21**, 269–274 (2011).

33. G. Mongillo, O. Barak, M. Tsodyks, Synaptic theory of working memory. *Science* **319**, 1543–1546 (2008).

34. D. Vandael, C. Borges-Merjane, X. Zhang, P. Jonas, Short-term plasticity at hippocampal mossy fiber synapses is induced by natural activity patterns and associated with vesicle pool engram formation. *Neuron* **107**, 509–521.e7 (2020).

35. V. Allen, P. Swigart, R. Cheung, S. Cockcroft, M. Katan, Regulation of inositol lipid-specific phospholipase cδ by changes in Ca^{2+} ion concentrations. *Biochem. J.* **327**, 545–552 (1997).

36. M. Nakahara, M. Shimozawa, Y. Nakamura, Y. Irino, M. Morita, Y. Kudo, K. Fukami, A novel phospholipase C, PLCη2, is a neuron-specific isozyme. *J. Biol. Chem.* **280**, 29128–29134 (2005).

37. D. Wang, L. Maler, Differential roles of Ca^{2+}/calmodulin-dependent kinases in posttetanic potentiation at input selective glutamatergic pathways. *Proc. Natl. Acad. Sci. U.S.A.* **95**, 7133–7138 (1998).

38. F. Fiumara, C. Milanese, A. Corradi, S. Giovedi, G. Leitinger, A. Menegon, P. G. Montarolo, F. Benfenati, M. Ghirardi, Phosphorylation of synapsin domain A is required for post-tetanic potentiation. *J. Cell Sci.* **120**, 3228–3237 (2007).

39. J. S. Lee, W. K. Ho, S. H. Lee, Post-tetanic increase in the fast-releasing synaptic vesicle pool at the expense of the slowly releasing pool. *J. Gen. Physiol.* **136**, 259–272 (2010).

40. N. Korogod, X. Lou, R. Schneggenburger, Posttetanic potentiation critically depends on an enhanced Ca^{2+} sensitivity of vesicle fusion mediated by presynaptic PKC. *Proc. Natl. Acad. Sci. U.S.A.* **104**, 15923–15928 (2007).

41. D. H. Brager, X. Cai, S. M. Thompson, Activity-dependent activation of presynaptic protein kinase C mediates post-tetanic potentiation. *Nat. Neurosci.* **6**, 551–552 (2003).

42. J. S. Lee, M. H. Kim, W. K. Ho, S. H. Lee, Presynaptic release probability and readily releasable pool size are regulated by two independent mechanisms during posttetanic potentiation at the calyx of Held synapse. *J. Neurosci.* **28**, 7945–7953 (2008).

43. L. S. Eliot, E. R. Kandel, R. D. Hawkins, Modulation of spontaneous transmitter release during depression and posttetanic potentiation of Aplysia sensory-motor neuron synapses isolated in culture. *J. Neurosci.* **14**, 3280–3292 (1994).

44. O. Genc, O. Kochubey, R. F. Toonen, M. Verhage, R. Schneggenburger, Munc18-1 is a dynamically regulated PKC target during short-term enhancement of transmitter release. *eLife* **3**, e01715 (2014).

45. C. C. Wang, C. Weyrer, D. Fioravante, P. S. Kaeser, W. G. Regehr, Presynaptic short-term plasticity persists in the absence of PKC phosphorylation of Munc18-1. *J. Neurosci.* **41**, 7329–7339 (2021).

46. D. Fioravante, M. H. Myoga, M. Leitges, W. G. Regehr, Adaptive regulation maintains posttetanic potentiation at cerebellar granule cell synapses in the absence of calcium-dependent PKC. *J. Neurosci.* **32**, 13004–13009 (2012).

47. O. Voiculescu, P. Charnay, S. Schneider-Maunoury, Expression pattern of a Krox-20/Cre knock-in allele in the developing hindbrain, bones, and peripheral nervous system. *Genesis* **26**, 123–126 (2000).

48. Y. Han, P. S. Kaeser, T. C. Südhof, R. Schneggenburger, RIM determines Ca^{2+} channel density and vesicle docking at the presynaptic active zone. *Neuron* **69**, 304–316 (2011).

49. I. Augustin, A. Betz, C. Herrmann, T. Jo, N. Brose, Differential expression of two novel Munc13 proteins in rat brain. *Biochem. J.* **337**, 363–371 (1999).

50. A. Betz, U. Ashery, M. Rickmann, I. Augustin, E. Neher, T. C. Südhof, J. Rettig, N. Brose, Munc13-1 is a presynaptic phorbol ester receptor that enhances neurotransmitter release. *Neuron* **21**, 123–136 (1998).

51. X. Lou, V. Scheuss, R. Schneggenburger, Allosteric modulation of the presynaptic Ca^{2+} sensor for vesicle fusion. *Nature* **435**, 497–501 (2005).

52. T. Sakaba, E. Neher, Quantitative relationship between transmitter release and calcium current at the calyx of Held synapse. *J. Neurosci.* **21**, 462–476 (2001).

53. M. Jang, E. Gould, J. Xu, E. J. Kim, J. H. Kim, Oligodendrocytes regulate presynaptic properties and neurotransmission through BDNF signaling in the mouse brainstem. *eLife* **8**, e42156 (2019).

54. Z. Chen, B. Cooper, S. Kalla, F. Varoqueaux, S. M. Young Jr., The Munc13 proteins differentially regulate readily releasable pool dynamics and calcium-dependent recovery at a central synapse. *J. Neurosci.* **33**, 8336–8351 (2013).

55. X. Lou, N. Korogod, N. Brose, R. Schneggenburger, Phorbol esters modulate spontaneous and Ca^{2+}-evoked transmitter release via acting on both Munc13 and protein kinase C. *J. Neurosci.* **28**, 8257–8267 (2008).

56. M. Ester, H.-P. Kriegel, J. Sander, X. Xiaowei, in *Proceedings of the Second International Conference on Knowledge Discovery in Databases and Data Mining* (AAAI Press, 1996), pp. 226–231.

57. B. Huang, S. A. Jones, B. Brandenburg, X. Zhuang, Whole-cell 3D STORM reveals interactions between cellular structures with nanometer-scale resolution. *Nat. Methods* **5**, 1047–1052 (2008).

58. B. Huang, W. Wang, M. Bates, X. Zhuang, Three-dimensional super-resolution imaging by stochastic optical reconstruction microscopy. *Science* **319**, 810–813 (2008).

59. N. Lipstein, S. Chang, K. H. Lin, F. J. Lopez-Murcia, E. Neher, H. Taschenberger, N. Brose, Munc13-1 is a Ca^{2+}-phospholipid-dependent vesicle priming hub that shapes synaptic short-term plasticity and enables sustained neurotransmission. *Neuron* **109**, 3980–4000.e7 (2021).

60. I. Weichard, H. Taschenberger, F. Gsell, G. Bornschein, A. Ritzau-Jost, H. Schmidt, R. J. Kittel, J. Eilers, E. Neher, S. Hallermann, J. Nerlich, Fully-primed slowly-recovering vesicles mediate presynaptic LTP at neocortical neurons. *Proc. Natl. Acad. Sci. U.S.A.* **120**, e2305460120 (2023).

61. T. Miki, Y. Nakamura, G. Malagon, E. Neher, A. Marty, Two-component latency distributions indicate two-step vesicular release at simple glutamatergic synapses. *Nat. Commun.* **9**, 3943 (2018).

62. S. Hallermann, A. Fejtova, H. Schmidt, A. Weyhersmuller, R. A. Silver, E. D. Gundelfinger, J. Eilers, Bassoon speeds vesicle reloading at a central excitatory synapse. *Neuron* **68**, 710–723 (2010).

63. F. Doussau, H. Schmidt, K. Dorgans, A. M. Valera, B. Poulain, P. Isope, Frequency-dependent mobilization of heterogeneous pools of synaptic vesicles shapes presynaptic plasticity. *eLife* **6**, e28935 (2017).

64. E. Neher, N. Brose, Dynamically primed synaptic vesicle states: Key to understand synaptic short-term plasticity. *Neuron* **100**, 1283–1291 (2018).

65. E. Neher, H. Taschenberger, Non-negative matrix factorization as a tool to distinguish between synaptic vesicles in different functional states. *Neuroscience* **458**, 182–202 (2021).

66. L. Y. Wang, L. K. Kaczmarek, High-frequency firing helps replenish the readily releasable pool of synaptic vesicles. *Nature* **394**, 384–388 (1998).

67. J. S. Dittman, W. G. Regehr, Calcium dependence and recovery kinetics of presynaptic depression at the climbing fiber to Purkinje cell synapse. *J. Neurosci.* **18**, 6147–6162 (1998).

68. H. Yang, M. A. Xu-Friedman, Relative roles of different mechanisms of depression at the mouse endbulb of Held. *J. Neurophysiol.* **99**, 2510–2521 (2008).

69. Y. Wang, P. B. Manis, Short-term synaptic depression and recovery at the mature mammalian endbulb of Held synapse in mice. *J. Neurophysiol.* **100**, 1255–1264 (2008).

70. J. S. Lee, W. K. Ho, E. Neher, S. H. Lee, Superpriming of synaptic vesicles after their recruitment to the readily releasable pool. *Proc. Natl. Acad. Sci. U.S.A.* **110**, 15079–15084 (2013).

71. N. Lipstein, T. Sakaba, B. H. Cooper, K. H. Lin, N. Strenzke, U. Ashery, J. S. Rhee, H. Taschenberger, E. Neher, N. Brose, Dynamic control of synaptic vesicle replenishment and short-term plasticity by Ca^{2+}-calmodulin-Munc13-1 signaling. *Neuron* **79**, 82–96 (2013).

72. F. Michelassi, H. Liu, Z. Hu, J. S. Dittman, A C1-C2 module in Munc13 inhibits calcium-dependent neurotransmitter release. *Neuron* **95**, 577–590.e5 (2017).

73. K. Grushin, R. V. Kalyana Sundaram, C. V. Sindelar, J. E. Rothman, Munc13 structural transitions and oligomers that may choreograph successive stages in vesicle priming for neurotransmitter release. *Proc. Natl. Acad. Sci. U.S.A.* **119**, e2121259119 (2022).

74. J. Xu, M. Camacho, Y. Xu, V. Esser, X. Liu, T. Trimbuch, Y. Z. Pan, C. Ma, D. R. Tomchick, C. Rosenmund, J. Rizo, Mechanistic insights into neurotransmitter release and presynaptic plasticity from the crystal structure of Munc13-1 C1C2BMUN. *eLife* **6**, e22567 (2017).

75. O. H. Shin, J. Lu, J. S. Rhee, D. R. Tomchick, Z. P. Pang, S. M. Wojcik, M. Camacho-Perez, N. Brose, M. Machius, J. Rizo, C. Rosenmund, T. C. Südhof, Munc13 C2B domain is an activity-dependent Ca^{2+} regulator of synaptic exocytosis. *Nat. Struct. Mol. Biol.* **17**, 280–288 (2010).

76. A. H. Tang, H. Chen, T. P. Li, S. R. Metzbower, H. D. MacGillavry, T. A. Blanpied, A trans-synaptic nanocolumn aligns neurotransmitter release to receptors. *Nature* **536**, 210–214 (2016).

77. K. Sätzler, L. F. Sohl, J. H. Bollmann, J. G. Borst, M. Frotscher, B. Sakmann, J. H. Lübke, Three-dimensional reconstruction of a calyx of Held and its postsynaptic principal neuron in the medial nucleus of the trapezoid body. *J. Neurosci.* **22**, 10567–10579 (2002).

78. H. Taschenberger, R. M. Leao, K. C. Rowland, G. A. Spirou, H. von Gersdorff, Optimizing synaptic architecture and efficiency for high-frequency transmission. *Neuron* **36**, 1127–1143 (2002).

79. J. G. Borst, J. Soria van Hoeve, The calyx of Held synapse: From model synapse to auditory relay. *Annu. Rev. Physiol.* **74**, 199–224 (2012).

80. M. Baydyuk, X. S. Wu, L. He, L. G. Wu, Brain-derived neurotrophic factor inhibits calcium channel activation, exocytosis, and endocytosis at a central nerve terminal. *J. Neurosci.* **35**, 4676–4682 (2015).

81. Y. Wu, Q. Liu, B. Guo, F. Ye, J. Ge, L. Xue, BDNF activates postsynaptic TrkB receptors to induce endocannabinoid release and inhibit presynaptic calcium influx at a calyx-type synapse. *J. Neurosci.* **40**, 8070–8087 (2020).

82. L. Li, H. Liu, Q. Hall, W. Wang, Y. Yu, J. M. Kaplan, Z. Hu, A hyperactive form of unc-13 enhances Ca^{2+} sensitivity and synaptic vesicle release probability in *C. elegans*. *Cell Rep.* **28**, 2979–2995.e4 (2019).

83. H. Liu, L. Li, J. Wang, J. Hu, J. Xia, X. Yu, J. Tang, H. Liu, X. Yang, C. Ma, L. Kang, Z. Hu, Mechanisms that regulate the C1-C2B mutual inhibition control functional switch of UNC-13. *eLife* **14**, RP105199 (2025).

84. M. Jusyte, N. Blaum, M. A. Bohme, M. M. M. Berns, A. E. Bonard, A. B. Vamosi, K. V. Pushpalatha, J. R. L. Kobbersmed, A. M. Walter, Unc13A dynamically stabilizes vesicle priming at synaptic release sites for short-term facilitation and homeostatic potentiation. *Cell Rep.* **42**, 112541 (2023).

85. W. Song, R. Ranjan, K. Dawson-Scully, P. Bronk, L. Marin, L. Seroude, Y. J. Lin, Z. Nie, H. L. Atwood, S. Benzer, K. E. Zinsmaier, Presynaptic regulation of neurotransmission in Drosophila by the G protein-coupled receptor methuselah. *Neuron* **36**, 105–119 (2002).

86. N. Blaum, T. Ghelani, T. W. B. Gotz, K. S. Chronister, M. Bengochea, L. Ceresnova, C. F. Christensen, T. C. Moulin, H. Kern, U. Thomas, M. Heine, S. J. Sigrist, A. M. Walter,

Monoamine-induced diacylglycerol signaling rapidly accumulates Unc13 in nanoclusters for fast presynaptic potentiation. *Proc. Natl. Acad. Sci. U.S.A.* **122**, e2514151122 (2025).

87. X. Liu, A. B. Seven, M. Camacho, V. Esser, J. Xu, T. Trimbuch, B. Quade, L. Su, C. Ma, C. Rosenmund, J. Rizo, Functional synergy between the Munc13 C-terminal C1 and C2 domains. *eLife* **5**, e13696 (2016).

88. R. Asadollahi, A. Ahmad, P. Boonsawat, J. Shahanoor Hinzen, M. Lohse, B. Bouazza-Arostegui, S. Sun, T. Utesch, J. D. Sommer, D. Ilic, M. Padmanarayana, K. Fischermanns, M. Ranjan, M. Boll, C. Ka, A. Piton, F. Mattioli, B. Isidor, K. Ounap, K. Reinson, M. H. Wojcik, C. R. Marshall, S. Mercimek-Andrews, N. Matsumoto, N. Miyake, B. O. Stephan, R. S. Honjo, D. R. Bertola, C. A. Kim, R. Yusupov, H. C. Mefford, J. Christodoulou, J. Lee, O. Heath, N. J. Brown, N. Baker, Z. Stark, M. Delatycki, N. J. Lake, S. Zeidler, L. Zuurbier, S. M. Maas, C. C. de Kruiff, F. Rajabi, L. H. Rodan, S. A. Coury, K. Platzer, H. Oppermann, R. Abou Jamra, S. Beblo, C. Maxton, R. Smigiel, H. Underhill, H. Dubbs, A. Rosen, K. L. Helbig, I. Helbig, S. M. Ruggiero, M. P. Fitzgerald, D. Kraemer, C. E. Prada, J. Tenney, P. Jayakar, S. Redon, J. Lefranc, K. Uguen, S. Race, S. Efthymiou, R. Maroofian, H. Houlden, S. Coppens, N. Deconinck, B. Ashokkumar, P. Varalakshmi, K. V. Gowda, F. Eghbal, E. Ghayoor Karimiani, M. Heidari, J. Neidhardt, M. Owczarek-Lipska, G. C. Korenke, M. J. Bamshad, P. M. Campeau, A. Lehman, L. G. Hendon, I. M. Wentzensen, K. G. Monaghan, Y. Chen, A. Szuto, R. D. Cohn, P. Y. B. Au, C. Hubner, F. Boschann, K. Manickam, D. C. Koboldt, A. Rad, G. Oprea, K. K. Bachman, A. H. Seeley, E. Agolini, A. Terracciano, P. Carmelo, C. Bupp, B. Grysko, A. Rein-Rothschild, B. Ben Zeev, A. Margolin, J. Morrison, A. Dagli, E. Stolerman, R. J. Louie, C. Washington, S. J. C. Stevens, M. Heijligers, F. S. Alkuraya, J. Lisfeld, A. Neu, F. Paoli Monteiro, A. L. Santos Pessoa, A. E. Camelo-Filho, F. Kok, D. Koeberl, K. Riley, L. Burglen, D. Doummar, B. Heron, C. Mignot, B. Keren, P. Charles, C. Nava, F. P. Bernhard, A. A. Kuhn, S. Thoms, R. D. Morrie, S. Mekhoubad, E. M. Green, S. J. Barmada, A. D. Gitler, O. Jahn, J. S. Rhee, C. Rosenmund, M. Mitkovski, H. Sticht, H. Sun, G. Le Gac, H. Taschenberger, N. Brose, J. S. Dittman, A. Rauch, N. Lipstein, Pathogenic UNC13A variants cause a neurodevelopmental syndrome by impairing synaptic function. *Nat. Genet.* **57**, 2691–2704 (2025).

89. M. Camacho, B. Quade, T. Trimbuch, J. Xu, L. Sari, J. Rizo, C. Rosenmund, Control of neurotransmitter release by two distinct membrane-binding faces of the Munc13-1 C_1C_2B region. *eLife* **10**, e72030 (2021).

90. D. M. Ramirez, E. T. Kavalali, Differential regulation of spontaneous and evoked neurotransmitter release at central synapses. *Curr. Opin. Neurobiol.* **21**, 275–282 (2011).

91. P. S. Kaeser, W. G. Regehr, Molecular mechanisms for synchronous, asynchronous, and spontaneous neurotransmitter release. *Annu. Rev. Physiol.* **76**, 333–363 (2014).

92. O. Kochubey, N. Babai, R. Schneggenburger, A synaptotagmin isoform switch during the development of an identified CNS synapse. *Neuron* **90**, 984–999 (2016).

93. Y. Tang, R. S. Zucker, Mitochondrial involvement in post-tetanic potentiation of synaptic transmission. *Neuron* **18**, 483–491 (1997).

94. C. Papantoniou, U. Laugks, J. Betzin, C. Capitanio, J. J. Ferrero, J. Sanchez-Prieto, S. Schoch, N. Brose, W. Baumeister, B. H. Cooper, C. Imig, V. Lucic, Munc13- and SNAP25-dependent molecular bridges play a key role in synaptic vesicle priming. *Sci. Adv.* **9**, eadf6222 (2023).

95. G. B. Awatramani, G. D. Price, L. O. Trussell, Modulation of transmitter release by presynaptic resting potential and background calcium levels. *Neuron* **48**, 109–121 (2005).

96. L. Y. Wang, E. Neher, H. Taschenberger, Synaptic vesicles in mature calyx of Held synapses sense higher nanodomain calcium concentrations during action potential-evoked glutamate release. *J. Neurosci.* **28**, 14450–14458 (2008).

97. Y. M. Yang, A. Fekete, J. Arsenault, A. S. Sengar, J. Aitoubah, G. Grande, A. Li, E. W. Salter, A. Wang, M. D. Mark, S. Herlitze, S. E. Egan, M. W. Salter, L. Y. Wang, Intersectin-1 enhances calcium-dependent replenishment of the readily releasable pool of synaptic vesicles during development. *J. Physiol.* **603**, 6185–6206 (2025).

98. T. Sakaba, N. L. Kononenko, J. Bacetic, A. Pechstein, J. Schmoranzer, L. Yao, H. Barth, O. Shupliakov, O. Kobler, K. Aktories, V. Haucke, Fast neurotransmitter release regulated by the endocytic scaffold intersectin. *Proc. Natl. Acad. Sci. U.S.A.* **110**, 8266–8271 (2013).

99. T. Butola, C. Wichmann, T. Moser, Piccolo promotes vesicle replenishment at a fast central auditory synapse. *Front. Synaptic Neurosci.* **9**, 14 (2017).

100. D. J. Weingarten, A. Shrestha, K. Juda-Nelson, S. A. Kissiwaa, E. Spruston, S. L. Jackman, Fast resupply of synaptic vesicles requires synaptotagmin-3. *Nature* **611**, 320–325 (2022).

101. C. Keine, M. Al-Yaari, T. Radulovic, C. I. Thomas, P. Valino Ramos, D. Guerrero-Given, M. Ranjan, H. Taschenberger, N. Kamasawa, S. M. Young Jr., Presynaptic Rac1 controls synaptic strength through the regulation of synaptic vesicle priming. *eLife* **11**, e81505 (2022).

102. D. R. Dries, L. L. Gallegos, A. C. Newton, A single residue in the C1 domain sensitizes novel protein kinase C isoforms to cellular diacylglycerol production. *J. Biol. Chem.* **282**, 826–830 (2007).

103. T. Virmani, M. Ertunc, Y. Sara, M. Mozhayeva, E. T. Kavalali, Phorbol esters target the activity-dependent recycling pool and spare spontaneous vesicle recycling. *J. Neurosci.* **25**, 10922–10929 (2005).

104. C. Y. Chang, X. Jiang, K. L. Moulder, S. Mennerick, Rapid activation of dormant presynaptic terminals by phorbol esters. *J. Neurosci.* **30**, 10048–10060 (2010).

105. S. De, C. F. Shuler, J. E. Turman Jr., The ontogeny of Krox-20 expression in brainstem and cerebellar neurons. *J. Chem. Neuroanat.* **25**, 213–226 (2003).

106. M. Lakso, J. G. Pichel, J. R. Gorman, B. Sauer, Y. Okamoto, E. Lee, F. W. Alt, H. Westphal, Efficient in vivo manipulation of mouse genomic sequences at the zygote stage. *Proc. Natl. Acad. Sci. U.S.A.* **93**, 5860–5865 (1996).

107. M. F. Juette, T. J. Gould, M. D. Lessard, M. J. Mlodzianoski, B. S. Nagpure, B. T. Bennett, S. T. Hess, J. Bewersdorf, Three-dimensional sub-100 nm resolution fluorescence microscopy of thick samples. *Nat. Methods* **5**, 527–529 (2008).

108. M. J. Mlodzianoski, M. F. Juette, G. L. Beane, J. Bewersdorf, Experimental characterization of 3D localization techniques for particle-tracking and super-resolution microscopy. *Opt. Express* **17**, 8264–8277 (2009).

109. M. Ester, H. P. Kriegel, J. Sander, X. Xiaowei, paper presented at the *Conference: 2. International Conference on Knowledge Discovery and Data Mining*, Portland, OR, 2 to 4 August 1996.

110. T. Sakaba, Roles of the fast-releasing and the slowly releasing vesicles in synaptic transmission at the calyx of Held. *J. Neurosci.* **26**, 5863–5871 (2006).

111. E. Neher, Merits and limitations of vesicle pool models in view of heterogeneous populations of synaptic vesicles. *Neuron* **87**, 1131–1142 (2015).

112. F. Varoqueaux, A. Sigler, J. S. Rhee, N. Brose, C. Enk, K. Reim, C. Rosenmund, Total arrest of spontaneous and evoked synaptic transmission but normal synaptogenesis in the absence of Munc13-mediated vesicle priming. *Proc. Natl. Acad. Sci. U.S.A.* **99**, 9037–9042 (2002).

113. R. M. Leao, H. von Gersdorff, Synaptic vesicle pool size, release probability and synaptic depression are sensitive to Ca^{2+} buffering capacity in the developing rat calyx of Held. *Braz. J. Med. Biol. Res.* **42**, 94–104 (2009).
